# Amino acid metabolism in tumor biology and therapy

**DOI:** 10.1038/s41419-024-06435-w

**Published:** 2024-01-13

**Authors:** Jie Chen, Likun Cui, Shaoteng Lu, Sheng Xu

**Affiliations:** 1 National Key Lab of Immunity and Inflammation and Institute of Immunology, Naval Medical University/Second Military Medical University, Shanghai, 200433 China; 2 Shanghai Institute of Stem Cell Research and Clinical Translation, Shanghai, 200120 China

**Keywords:** Cancer, Cancer metabolism

## Abstract

Amino acid metabolism plays important roles in tumor biology and tumor therapy. Accumulating evidence has shown that amino acids contribute to tumorigenesis and tumor immunity by acting as nutrients, signaling molecules, and could also regulate gene transcription and epigenetic modification. Therefore, targeting amino acid metabolism will provide new ideas for tumor treatment and become an important therapeutic approach after surgery, radiotherapy, and chemotherapy. In this review, we systematically summarize the recent progress of amino acid metabolism in malignancy and their interaction with signal pathways as well as their effect on tumor microenvironment and epigenetic modification. Collectively, we also highlight the potential therapeutic application and future expectation.

## Facts

- Altered amino acid metabolism in tumors challenges the traditional classification of essential and nonessential amino acids.
- Amino acids have emerged as pivotal regulators in tumors, participated in a myriad of bidirectional interactions including signal pathways, tumor microenvironment, and epigenetic modifications.
- Clinical trials align with the idea that limiting amino acid intake may improve cancer prognoses.

## Open Questions

- Among the several effects that are simultaneously regulated by certain amino acid, is there a chief effect that determines the progression or repression of tumor?
- What are the optimal strategies and urgent challenges for the clinical translation of amino acids-based therapies in the near future?
- Is the altered amino acids metabolism, described in different tumors, causally linked to their tumor etiology and pathogenesis?

## Introduction

The ‘use of opportunistic modes of nutrient acquisition’ was recently described as a hallmark of cancer cells [1], living in a nutrient-poor microenvironment. Cancer cells must adapt their metabolism to support biomass production, ATP generation and maintain a redox state. Disrupting these processes can interfere with both tumor growth and proliferation [2]. Amino acids, like other biomacromolecules, play an important role in rapidly proliferating cancer cells, as carbon and nitrogen donors to get rid of the nutrition limitation [3]. Thus, amino acid metabolism has been extensively studied following glucose metabolism in tumor.

While the definition of essential amino acids (EAAs) and nonessential amino acids (NEAAs) is appropriate for normal cells, the classification does not apply to cancer cells [4]. Altered amino acids metabolism is common in tumors, and non-essential amino acids usually become essential in tumors. Thus, targeting certain kind of amino acids has the potential to control specific tumors. Targeting asparagine metabolism enzyme such as asparaginase have the potential to treat leukemia, which is currently in clinical use. Furthermore, targeting molecules in amino acid metabolic signaling pathway also has the potential to treat tumors [5]. For example, targeting mammalian target of rapamycin (mTOR) can control the growth of various tumors including breast cancer, kidney cancer, neuroendocrine cancer and so on. Other key molecules including MYC, and KRAS in amino acid metabolic signaling pathway are also burgeoning approaches for tumor biotherapy.

In addition to altered nutrient and signal pathway, solid tumors are known to recruit immune cells in the stroma and create favorable conditions for their growth and survival [6], which is known as tumor microenvironment (TME). Cells in TME could not only resist immune surveillance and drug therapy, but also provide amino acids to tumors to meet their growth needs. Thus, restricting amino acids in TME is an effective way to limit tumor growth [7]. Besides, amino acids also play an important role in epigenetics like DNA methylation and histone modification [8]. Improving our understanding of its role in tumor progression and immune evasion could provide novel ideas for metabolic cancer therapy.

## Reprogrammed amino acid metabolism in cancer

A number of cancers have been found auxotrophic for NEAAs [9]. It may be that the demand for proliferate is too large and exceed the supply, or the related enzymes are mutated, or metabolic pathways are dysregulated. These amino acids are named conditional EAAs. Considering the swift proliferation of tumors within an environment deprived of nutrients, the composition of amino acids frequently displays instability. This fluctuation in amino acids can significantly impact overall cellular metabolism, ultimately culminating in cell proliferation or death [10]. Therefore, the 20 standard proteinogenic amino acids, including conditional EAAs (glutamine, arginine), EAAs (branched-chain amino acids, tryptophan), and non-essential amino acids (asparagine, aspartate) play flexible roles in protein synthesis or energy supply activities in tumor.

### Glutamine metabolism

Glutamine (Gln) is a conditional EAA, which is not essential for normal cells but becomes crucial for tumor cells due to their heightened demand. It is the most abundant amino acid found in plasma, and the most rapidly consumed amino acid in tumor cells [11]. As an EAA in tumors, glutamine participates in rapid biosynthetic reactions in tumor. Tumor cells utilize glutamine avidly, known as glutamine addiction [12]. Thus, it always functions as the rate-limiting molecule of the cell reproductive cycle. Once deprived of glutamine, cancer cells usually arrest in S-phase [13]. Additionally, glutamine also plays a crucial role in maintaining redox homeostasis, replenishing the tricarboxylic acid (TCA) cycle, and participating in signal transduction processes within tumors [14].

**Fig. 1:**
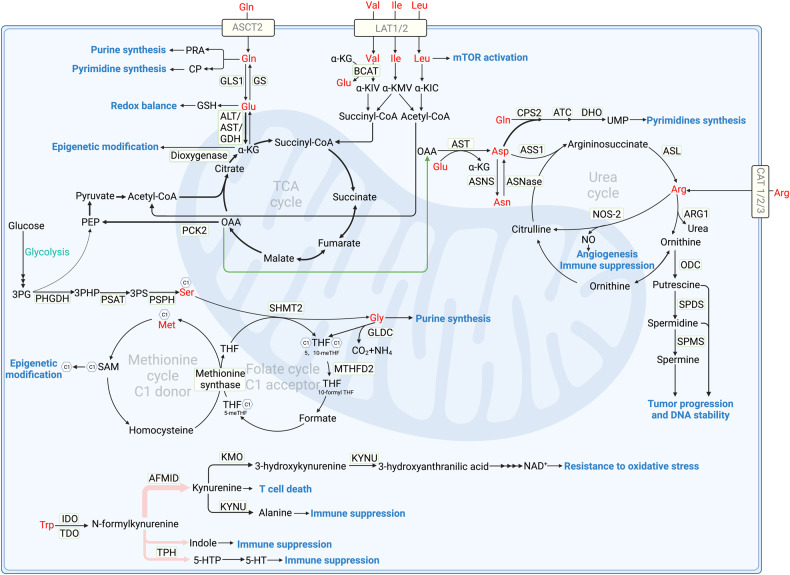
Amino acid metabolism in tumors. ASCT2 serves as the primary glutamine transporter, importing glutamine to generate purines and pyrimidines. GLS1 catalyzes glutaminolysis, generating glutamate which synthesizes GSH to maintain redox balance. Glutamate further transfers to α-KG under the catalyze of transaminase or deaminase. Under glucose-limited condition, glutamate derived α-KG acts as an alternative for glucose through participating in TCA cycle. Elevated PCK2 prompts PEP production to fuel TCA cycle. α-KG also act as substrate for DNA dioxygenase in demethylation. LAT1/2 transports BCAAs intracellular, activating mTOR activity and producing BCKA under the catalyze of BCAT2. BCKA can be further catalyzed to acetyl-CoA and succinyl-CoA to fuel TCA cycle. Arginine can be obtained through CAT family transporters, and synthesized de novo under the catalysis of ASS1 and ASL from citrulline in urea cycle. In tumors, ornithine is metabolized by upregulated ODC into polyamines including putrescine, spermidine, and spermine. The overproduction of polyamines results in uncontrolled tumor growth. Besides, NO production under the catalyze of NOS-2 elevates angiogenesis and suppress immune. De novo serine synthesis is catalyzed by PHGDH, PSAT and PSPH. SHMT2 catalyzes glycine and 5,10-methylene-THF production, in which glycine can also be transferred to 5,10-methylene-THF under the catalyze of GLDC, participating in folate cycle and methionine cycle. Besides, glycine also directly supplies carbon for de novo purine biosynthesis. Tryptophan is mainly catalyzed by IDO1 and TDO to produce kynurenine, and finally generates NAD^+^ and alanine to inhibit immune response and promote cancer progression. Seldom tryptophan metabolizes along the 5-HT and indole pathway, also function to suppress immune response. GLS1 Glutaminase1, GS Glutamine synthetase, GOT Glutamic oxaloacetic transaminase, GPT Glutamic pyruvic transaminase, GDH Glutamate dehydrogenase, α-KG α-ketoglutarate, OAA Oxaloacetic acid, PCK2 Phosphoenolpyruvate carboxykinase2, PEP Phosphoenolpyruvate, CP Carbamoyl phosphate, PRA Phosphoribosyl amine, ASNS Asparagine synthase, ASNase Asparaginase, CPS carbamoyl phosphate synthase, ATC Aspartate transcarbamylase, DHO Dihydroorotase, ASS1 Argininosuccinate synthase1, ASL Argininosuccinate lyase, ARG1 Arginase1, NOS-2 Nitric oxide synthase-2, ODC Ornithine decarboxylase, SPDS Spermidine synthase, SPMS Spermine synthase, 3PG 3-phosphoglyceric acid, 3PHP 3-phosphohydroxypyruvate, 3PS 3-phosphoserine, PHGDH Phosphoglycerate dehydrogenase, PSAT Phosphoserine aminotransferase, PSPH Phosphoserine phosphatase, SHMT2 Serine hydroxymethyltransferase2, GLDC Glycine dehydrogenase, MTHFD2 Methylenetetrahydrofolate dehydrogenase2, THF Tetrahydrofolate, SAM S-adenosyl methionine, IDO Indoleamine 2,3-dioxygenase, TDO Tryptophan 2,3-dioxygenase, AFMID arylformamidase, KMO Kynurenine 3-monooxygenase, KYNU Kynureninase, TPH Tryptophan hydroxylase, Gln Glutamine, Glu Glutamate, Val Valine, Ile Ileucine, Leu Leucine, Asp Asparate, Asn Asparagine, Arg Arginine, Ser Serine, Gly Glycine, Met Methionine, Trp Tryptophan.

ASCT2(SLC1A5) is the main glutamine transporter in tumor (Fig. 1). It is regulated by multiple tumor associated transcription factors including Rb/E2F [15], androgen receptor 3 [16] and ATF4 [17]. ASCT2 is highly expressed in tumor tissue, and its expression level is negatively correlated with patients’ prognosis. As ASCT2 transports glutamine for tumor consumption, inhibiting ASCT2 induces apoptosis and exhibits anti-cancer activity in acute myeloid leukemia [18], gastric cancer [19], prostate cancer [20], and triple-negative breast cancer [21]. In addition, tumor cells are capable of synthesize glutamine by themselves from glutamate (Glu) and ammonia. Glutamine synthetase (GS) is highly expressed in cancer cells to support their rapid proliferation. Moreover, GS can also promote cell proliferation independently of its catalytic function, only by interacting with nuclear pore protein [22]. Therefore, tumor cells acquire a substantial amount of glutamine through both intrinsically synthesis and extrinsically uptake, emphasizing its critical role in tumor reprogrammed metabolism.

Glutaminolysis is a process catalyzed by glutaminase 1(GLS1) or GLS2 to produce glutamate [23]. GLS1 and GLS2 are isozymes that play opposite roles in tumor development [24]. GLS1 has oncogenic properties, while GLS2 has been described as a tumor suppressor. Numerous studies have reported a higher expression of GLS1 and lower expression of GLS2 in various tumor types, including liver cancer and colorectal cancer [25, 26]. GLS1 is regulated by the oncogenes MYC [27], Rho GTPases [28] and Notch [29]. In colorectal cancer cells, GLS1 is essential to tumor growth, invasion, and metastatic colonization. Mechanically, under hypoxia TME, HIF-1 activates the expression of the GLS1 to promote tumor migration, invasion, and metastatic colonization [26]. Besides, GLS1 plays a crucial role in boosting the production of GSH and NADH, contributing to oxidative balance maintain to promote tumor proliferation [30]. Conversely, GLS2 expression is transcriptionally upregulated by tumor suppressor and stress-related proteins, including p53, p63, and p73. Although GLS2 plays a role in the production of GSH, it is worth noticing that GLS2 modestly regulates the ratio of GSH/GSSG, which is essential to oxidative balance. Different from GLS1, GLS2 catalyzes glutamate metabolism to promote α-ketoglutarate (α-KG) production, participating in TCA cycle and thereby facilitating the production of lipid ROS. The accumulation of ROS leads to mitochondrial membrane hyperpolarization and thereby inducing ferroptosis [25]. In addition to glutaminolysis, glutamine can be metabolized into intermediates like carbamoyl phosphate (CP) and phosphoribosyl amine (PRA) for the synthesis of purines and pyrimidines, which are essential components for DNA synthesis and repair during rapidly tumor proliferation [31, 32].

Glutamate, the metabolite of glutaminolysis, provides important resources for energy and biomacromolecule synthesis in tumor (Fig. 1). In tumors under glucose-limited condition, glutamate acts as a substitute for glucose, producing the intermediate α-KG to facilitate the TCA cycle. However, the provision of α-KG alone is inadequate for sustaining the TCA cycle. This insufficiency arises from the limited availability of acetyl-CoA, a rate-limiting molecule in TCA cycle. Studies have found that in glutamine addictive tumor, mitochondrial phosphoenolpyruvate carboxykinase (PCK2) expression was elevated, facilitating phosphoenolpyruvate (PEP) production from oxaloacetate generated in the TCA cycle [33]. Thus, glutamine-derived PEP acts as a substitute for glucose-derived PEP, offering a valuable source to acetyl-CoA production that replenishes the TCA cycle [5]. Glutamate and α-KG also participate in the transaminate to other non-essential amino acids. In addition, α-KG serves as both substrate and cofactor for DNA dioxygenase enzymes involved in DNA demethylation [34] (Detailed later in amino acid metabolism and epigenetic modification).

### Arginine metabolism

Arginine (Arg) is also identified as a conditional EAA in tumor [5]. It can be synthesized de novo under the catalysis of argininosuccinate synthase 1 (ASS1) and argininosuccinate lyase (ASL) from aspartate and citrulline in urea cycle (Fig. 1). However, ASS1, the rate-limiting enzyme in urea cycle, is usually downregulated in cancer and its downregulation has been reported to be associated with advanced tumor stage [35]. ASS1 downregulation redirects aspartate from urea production towards pyrimidine biosynthesis, facilitating the high demand of rapid tumor proliferation. This phenomenon is commonly known as urea cycle dysregulation [36]. Since tumors underexpress urea cycle related enzymes like ASS1 and therefore downregulate arginine synthesis, exogenous arginine supply is critical for tumors survival and proliferation. Arginine can be obtained through cationic amino acid transporter (CAT) family transporters, including CAT-1, CAT-2 and CAT-3. CATs are always upregulated in many human cancers [37]. Thus, in arginine-dependent cancer cells, CATs knockdown can decrease the viability of cancer cells and induce apoptosis [38].

Arginine can be hydrolyzed by arginases (cytoplasmic, ARG1; mitochondrial, ARG2) and both arginases are upregulated in cancer cells to ensure the production of polyamines. ARG1 is upregulated in a wider range of tumors compared to ARG2. ARGs convert arginine to urea and ornithine. In tumors, ornithine is metabolized by upregulated ornithine decarboxylase (ODC) into polyamines including putrescine, spermidine, and spermine [39]. Polyamines are well known for their crucial role in tumor proliferation and DNA stability [40–42]. They facilitate cell proliferation by increasing DNA synthesis through the activation of enzymes such as DNA polymerases, helicases, and DNA ligases. Besides, cellular protein synthesis is also positively correlated with polyamines. Moreover, natural polyamines function as free radical scavengers. Thus, the strong affinity between polyamines and DNA enables the stabilization of DNA structure, granting polyamines the capacity to protect nucleic acids from damage [43, 44].

In addition, arginine can produce NO under the catalysis of nitric oxide synthase-2 (NOS-2) in tumor and macrophage. NO affects TME and tumor proliferation by promoting angiogenesis [45]. Besides, NO derived peroxynitrite can nitrate tyrosine residues and block tyrosine protein phosphorylation, reducing T cell proliferation and activation [46].

### Branched-chain amino acid (BCAA) metabolism

BCAAs, namely isoleucine (Ile), leucine (Leu), and valine (Val), are closely interconnected and classified as EAAs, whether in normal or tumor cells. Changes in the level of one of the BCAAs are accompanied by changes in the other two with the same direction and magnitude [2]. As EAAs, BCAAs cannot be synthesized in human and thus corresponding transporters are critical. LAT1(SLC7A5) and LAT2(SLC7A8) serve as primary transporters for BCAAs [47, 48] (Fig. 1), exhibiting high expression levels in glioblastoma and clear cell renal cell carcinoma [49, 50]. Drugs targeting LATs (BAY-8002, JPH203, OKY034 etc.) have already been used in preclinical treatment of cancer [51, 52].

BCAAs affect protein synthesis either by transmitting the signal of cell nutritional state or acting as proteinogenic amino acids [53]. BCAAs accumulation mainly promotes mTORC1 activition to enhance tumor development and growth [54]. Explicitly, mTORC1 triggers a cascade of signaling pathway through phosphorylating its downstream effectors, including eukaryotic translation initiation factor 4E binding protein 1(4EBP-1), p70 ribosomal S6 kinase 1 (S6K1), and sterol regulatory element binding protein (SREBP), to regulate autophagy and synthesize lipids, nucleotides and proteins [55] (Detailed later in signal pathways in amino acid metabolism). For another, BCAAs, especially leucine, are essential for protein synthesis as they are in great demand in new protein translation [56].

BCAAs catabolism and related enzymes are closely related to tumorigenesis. Leucine, isoleucine and valine catabolism is mediated by BCAA transaminase 2 (BCAT2) to produce branched chain α-keto acid (BCKA) including α-ketoisocaproate (α-KIC), α-ketoamethylvalerate (α-KMV) and α-ketoisovalerate (α-KIV) respectively. Subsequently, BCKAs like α-KIC can undergo further metabolic conversion into acetyl-CoA, while α-KIV can be metabolized into succinyl-CoA. As for α-KMV, it can undergo further metabolic conversion into both acetyl-CoA and succinyl-CoA. These metabolites actively participate in the TCA cycle. Thus, BCAAs catabolism is critical for the development of cancers, especially pancreatic ductal adenocarcinoma [54]. BCAAs also play a vital role in the synthesis of nucleotide through sustaining the levels of the enzyme ribonucleotide reductase regulatory subunit M2 (RRM2) [57, 58]. Since BCAAs are tightly related to tumors, altered BCAAs level in blood can predict the development of certain tumors in both humans and mouse [59].

### Tryptophan metabolism

Tryptophan (Trp) is also an EAA as its anabolism is absence in vivo. Tryptophan is involved in inherent malignant characteristic of tumors and can limit tumor immunity. The most important metabolic pathway for tryptophan is the kynurenine pathway. Free rather than albumin-binding tryptophan can be catalyzed by indoleamine 2,3-dioxygenase1 (IDO1) and tryptophan 2,3-dioxygenase (TDO) to produce kynurenine [60] (Fig. 1). Along the kynurenine pathway, a series of biologically active molecules are produced to influence tumor progression. The primary metabolite kynurenine has been reported to block T cell proliferation and induce T cell death [61]. Advanced cancers were associated with an increased kynurenine/tryptophan ratio, indicating that kynurenine level is correlated with tumor malignancy [62]. Under the catalyze of kynurenine 3-monooxygenase(KMO) and kynureninase(KYNU), kynurenine can be further catabolized to NAD^+^ and alanine. Kynurenine pathway is known as the de novo NAD^+^ synthesis pathway, exhibiting potent resistance to oxidative stress and promoting cancer cell metastasis [63]. In vivo studies have revealed that changes in tryptophan metabolism can decrease NAD^+^ synthesis and DNA damage, thereby promoting hepatocarcinogenesis [64]. Besides, alanine is deleterious for spheroid growth and thereby suppresses cancer progression [65].

Apart from kynurenine pathway, tryptophan can also be metabolized in 5-hydroxytryptamine (5-HT) pathway and indole pathway, accounting for less than 5% of tryptophan metabolism [66]. 5-HT is also called serotonin, which has more recently emerged as a growth factor for human tumor cells of different origins [67]. Serotonin enhanced expression of PD-L1 on mouse and human cancer cells in vitro via serotonylation, covalent bonds formation between glutamine residues and serotonin, resulting in tumor progression [68]. Along with the indole pathway, indole production activates the aryl hydrocarbon receptor (AhR) in tumor-associated macrophages (TAMs), and thus inhibits intratumoral CD8^+^ T cell function [69]. Tryptophan metabolism also plays a vital role in the TME, see below amino acid in TME for details.

Increased kynurenine pathway is correlated with tumor progression. In tumors such as non-small cell lung cancer (NSCLC) and esophageal squamous cell cancer, higher IDO1 and TDO expression in kynurenine pathway is associated with higher TNM stage and shorter overall survival [70]. IDO1 expression can be either triggered as a counter regulatory response to cytokines like IL-1β and IL-6 released from tumor-infiltrating immune cells or maintained through tumor-intrinsic oncogenic signaling [71, 72]. Studies found that intratumoural IDO1 expression has been shown to correlate with the frequency of liver metastases in colorectal cancer [73]. Besides, overexpression of IDO1 augmentes the motility of lung cancer cells, whereas its knockdown reduced cancer motility [74]. TDO, an enzyme that catalyzes the same reaction as IDO1, is also linked to a poor prognosis when overexpressed [66]. In a mouse model of lung cancer, inhibited TDO resulted in a reduction in the number of tumor nodules in the lungs [75].

### Asparagine and aspartate

Asparagine (Asn) and aspartate (Asp) are inter-convertible and are classified as non-essential amino acids, which play vital roles in tumor proliferation and metastasis. Asparagine synthase (ASNS) catalyzes aspartate to generate asparagine, while asparaginase (ASNase) catalyzes asparagine to produce aspartate (Fig. 1). Another way to produce aspartate is to use amino from glutamate and OAA from TCA cycle under the catalyze of glutamic oxaloacetic transaminase (GOT). Aspartate is the limiting metabolite for proliferation in tumors under hypoxia, which level is correlated with hypoxic markers [76]. Besides, aspartate has poor cell permeability, which prevents its environmental acquisition. Therefore, inhibited intracellular aspartate synthesis and limited extracellular aspartate uptake represse tumors proliferation [77]. However, asparagine can be efficiently imported into tumors. Tumors with high ASNase expression can rescue tumor suppression through conversion of asparagine into aspartate, bypassing intrinsic aspartate limitation and promoting tumor growth [78].

Asparagine and aspartate metabolism plays multiple roles in tumor progression. Aspartate originally participates in urea cycle. However, in many tumors, loss ASS1 in urea cycle promotes cancer proliferation by diversion of aspartate substrate towards carbamoyl-phosphate synthase 2 (CPS2), aspartate transcarbamylase (ATC), and dihydroorotase (DHO), enzymes that catalyze the first three reactions in the pyrimidine synthesis pathway, resulting in increased tumor progression [36]. Besides, asparagine export is accompanied by reverse transport of serine, arginine and histidine. Thus its intracellular level is critical for various amino acids uptake and therefore protein synthesis [79]. Proteomic studies have shown that asparagine is specifically enriched in proteins associated with epithelial-mesenchymal transition (EMT) and restricting its availability hampers the level of EMT associated proteins [80].

Despite regulating pyrimidine and EMT-related proteins synthesis to promote tumor progression, asparagine also regulates mesenchymal-epithelial transition (MET) to complete tumor colonization at distant metastatic sites [81]. Mechanically, the scarcity of glutamine at distant metastatic sites, coupled with the heightened bioavailability of asparagine within these sites, triggers the activation of GS [82]. This activation propels glutamine biosynthesis, fostering the accumulation of HIF1α and MYC, which are pivotal factors in metastatic processes. The relative abundance of asparagine and glutamine may thus have critical effects on tumor cells at metastatic sites. Besides, HIF1α and MYC are associated with increased oxidant stress and play important roles in the transition of EMT-like tumor cells to MET-like state, which is necessary for metastatic colonization. Thus, in an aggressive breast cancer model, ASNS upregulation promotes metastasis and results in the development of widespread metastases in the brain, liver, and lungs [80]. Consistently, asparagine restriction can repress above processes and prolong the survival of patients [40].

### Serine/glycine and one-carbon metabolism

As one-carbon donors in the folate cycle, serine, glycine and their associated enzymes significantly contribute to nucleotide synthesis, methylation reaction and redox homeostasis to promote tumor progression. Serine hydroxymethyltransferase (cytoplasmic, SHMT1; mitochondrial, SHMT2) catalyzes the transfer of carbon from serine to tetrahydrofolate (THF), resulting in the formation of 5,10-methylene-THF, which is essential for nucleotide synthesis to fuel rapid tumor proliferation (Fig. 1). Large-scale genomic study of human tumors reveals that SHMT2 is essential for cancer cell survival and its knockdown severely impairs cancer cell proliferation [83, 84]. Methylenetetrahydrofolate dehydrogenase2 (MTHFD2), acting as one-carbon metabolism related enzyme, is upregulated and associated folate cycle with methionine cycle to promote S-adenosyl methionine (SAM) production in tumor cells [85]. Thereby, serine/glycine metabolism contributes to methylation of genes and proteins as well as maintains redox homeostasis [86] (Detailed later in amino acid metabolism and epigenetic modification). Additionally, post-translational modifications of those metabolic enzymes also play a regulatory role in tumor metabolism and progression. Deacetylation of SHMT2 by SIRT3 promotes its enzymatic activity, increases serine consumption and finally promotes colorectal carcinogenesis [87]. When MTHFD2 is hyperacetylated, its enzymatic activity is inhibited and thereby NADPH level is downregulated. SIRT3 is also responsible for MTHFD2 deacetylation to maintain redox balance, which can be inhibited by cisplatin in colorectal cancer cells [88].

As a non-essential amino acid, the synthesis of serine is vital to tumors. De novo serine synthesis pathway (SSP) startes from 3-phosphoglyceric acid (3PG) generated from glycolysis, catalyzed by enzymes phosphoglycerate dehydrogenase (PHGDH), phosphoserine aminotransferase (PSAT), and phosphoserine phosphatase (PSPH). As the rate-limiting enzyme, tumor cells highly express PHGDH to counteract limited serine availability [89]. Conversely, RNF5, an E3 ubiquitin ligase, mediates PHGDH degradation and suppresses tumor progression [89, 90]. With enough serine, serine palmitoyltransferase (SPT) catalyzes the de novo biosynthesis of sphingolipids. However, when serine synthesis is limited, SPT will instead use alanine as a substrate to synthesize cytotoxic deoxysphingolipids and then suppress tumor [65]. Thus, maintaining certain level of serine is necessary for tumor cells to escape from cytotoxic suppression.

Mitochondrial SHMT2 is the primary catalyst for glycine production from serine, thereby promoting the folate cycle. Elevated glycine level is linked to cancer progression like multiple myeloma (MM) and lymphoma [91]. The glycine concentration in the bone marrow is elevated due to bone collagen degradation mediated by MM cell-secreted matrix metallopeptidase 13 (MMP13) [92]. Although glycine is a non-essential amino acid, experiments have shown that limiting the supply of exogenous glycine induces tumor cells arrested in the growth phase (G1 phase). It is worth noting that glycine is required for nucleotide biosynthesis, directly supplying carbons for de novo purine biosynthesis, or donating one-carbon unit to the folate pool via the mitochondrial glycine cleavage system under the catalyze of glycine dehydrogenase (GLDC) [93]. Besides, remodeled glycine metabolism mediated by protein arginine methyltransferase 7 (PRMT7) induces toxic death of leukemia stem cells [12]. Mechanistically, PRMT7 loss resulted in reduced expression of glycine decarboxylase, leading to the reprogramed glycine metabolism to generate methylglyoxal, which is detrimental to leukemia stem cells.

## Signal pathways in amino acid metabolism

Conventional understanding states amino acids as essential building blocks for peptide and protein synthesis. However, recent research has shed light on the profound significance of amino acids as bioactive molecules that play active roles in signaling pathways and metabolic regulation. mTOR, MYC and KRAS, which sense cellular amino acid levels and orchestrate these signals in a coordinated manner, play crucial roles in maintaining cellular metabolic homeostasis. Importantly, not only changes in amino acid levels impact signal pathways, but alterations in signaling pathways can also affect amino acid metabolism.

### mTOR senses and regulates amino acid metabolism

mTOR is an atypical serine/threonine protein kinase, acting as a convergence point for anabolism and catabolism. Due to differences in structure and function, mTOR complexes are categorized as mTORC1 and mTORC2. mTORC1, which is sensitive to rapamycin inhibition, comprises mTOR, Raptor, mLST8, Tti/Tel2 and suppressive subunits PRAS40 and Deptor. The phosphorylation of PRAS40 and Deptor relieves its inhibition and activates mTORC1 [94]. 4EBP-1, S6K1 and SREBP are downstream effectors of mTORC1, which are associated with upregulated synthesis as well as poor prognosis in cancer [95]. mTORC1 is negatively regulated by low energy conditions, hypoxia, and DNA damage. It is also positively regulated by growth factors like insulin/insulin-like growth factor-1 (IGF-1) pathway and receptor tyrosine kinase-dependent Ras signaling. Particularly, when amino acids are abundant, the mTORC1 signaling pathway is positively regulated to transmit signals to facilitate protein synthesis. Conversely, under condition of amino acid insufficiency, the translation of proteins is inhibited to meet energy demand. Considering that cancer cells often exist in a nutrient-deficient environment, mTORC1 is consistently negatively regulated to adapt to metabolic alterations. mTORC2, which is insensitive to rapamycin, consists of mTOR, mSIN1, mLST8, Tti/Tel2 and suppressive subunit Rictor and Deptor. The balance between mTORC1 and mTORC2 orchestrates various metabolic processes, although our understanding of mTORC2 remains limited [96]. We mainly focus on the function of mTORC1 below.

**Fig. 2:**
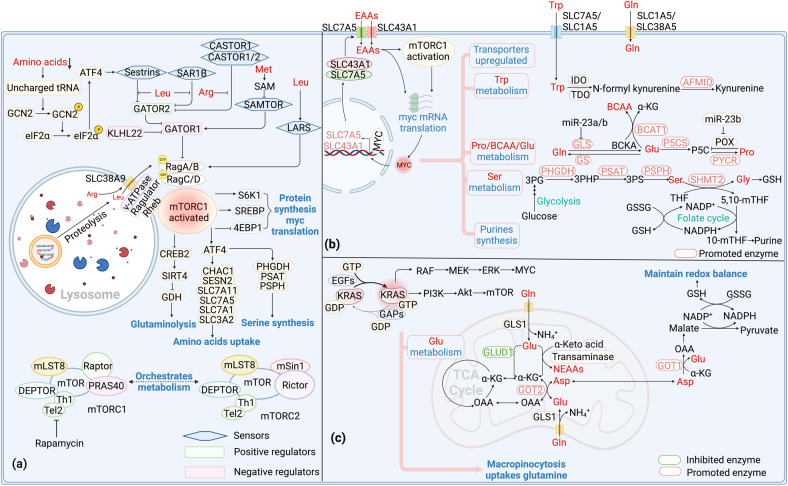
Signal pathways in amino acid metabolism. **a** mTOR signaling and amino acid metabolism. When amino acids are insufficient, GCN2 is activated by the uncharged tRNA and thereby leading to the activation of eIF2α and subsequently the upregulation of sestrins expression to regulate mTORC1 activity. Sestrins and SAR1B could be activated under leucine deprivation circumstance. After activation, sestrins and SAR1B bind and inhibit GATOR2, a positive regulator of mTORC1. In arginine-depleted condition, CASTOR1/2 forms dimers to inhibit mTORC1 activity by downregulating GATOR2 and upregulating GATOR1. SAMTOR senses methionine concentration in the form of SAM. When the concentration of methionine is down regulated, SAMTOR binds GATOR1 to inhibit mTORC1. LARS senses leucine concentration and interacts with Rag by directly mediating leucylation of RagA/B to activate mTORC1. Rag promotes mTORC1 to locate to lysosome which contains its activator Rheb. Ragulator provides a platform for lysosome to tether Rag. Besides, SLC38A9 participates in the activation of mTORC1 by influencing Rag. After activation, mTORC1 activates ATF4, stimulating serine synthesis and amino acids uptake. mTORC1 also promotes glutamine anaplerosis through regulating CREB2, SIRT4 and GDH. Furthermore, mTORC1 triggers phosphorylate 4EBP-1 and S6K1 to regulate protein synthesis and MYC translation. **b** MYC signaling and amino acid metabolism. MYC upregulates SLC7A5 and SLC43A1 to transport EAAs. These EAAs activate mTORC1 and Myc, forming a positive feedback loop. Meanwhile, SLC1A5 and SLC38A5 are also upregulated by MYC to transport NEAAs. Specifically, MYC promotes the conversion of tryptophan to kynurenine through inducing AFMID in the kynurenine pathway. MYC also upregulates GS to promote glutamine synthesis. Besides, MYC participates in glutamine catabolism by inducing miR-23a/b to suppress the expression of GLS. MYC induces P5CS and PYCR to promote proline synthesis. Meanwhile, it increases miR-23b to decrease the expression of POX/PRODH to inhibit proline catabolism. MYC upregulates BCAT1 and increases BCAAs synthesis. In addition, MYC can promote serine synthesis from glucose. It also upregulates SHMT2 to metabolize serine, producing glycine to participate in GSH production and 5,10-mTHF to participate in purine synthesis. **c** KRAS signaling and amino acid metabolism. Binding of KRAS-GTP to RAF stimulates its dimerization and activation, triggering the activation of MEK and ERK to drive cell cycle progression and proliferation. Binding of KRAS-GTP to PI3K activates AKT and mTOR, regulating apoptosis, metabolism and translation. Macropincytosis maintains intracellular glutamine levels following *Kras* activation. KRAS downregulates GLUD1, leading to alterations in glutamine metabolism to produce NEAAs instead of participating in TCA cycle. KRAS not only upregulates GOT1 but also upregulates GOT2, catabolizing glutamine-derived aspartate in mitochondria and aspartate into OAA in the cytoplasm and finally converted to pyruvate, resulting GSH production to maintain the cellular redox balance. mTOR Mammalian target of rapamycin, GCN2 General control nonderepressible2, LARS Leucyl-tRNA synthetase, S6K1 P70 ribosomal S6 kinase 1, 4EBP1 4E binding protein 1, SREBP Sterol regulatory element binding protein, CREB2 cAMP-responsive element binding 2, POX Proline oxidase, P5CS P5C synthase, PYCR P5C reductase, P5C Pyrroline-5-carboxylate, BCKA Branched chain α-keto acid, GSH Glutathione, GSSG Glutathione disulfide, GEFs Guanine nucleotide exchange factors, GAPs GTPase-activating proteins, GLUD1 Glutamate dehydrogenase1 EAAs Essential amino acids, NEAAs Non-essential amino acids, Pro Proline, Gly Glycine.

Amino acid sensors in cytoplasm like sestrins, SAR1B, CASTOR1/2, SAMTOR and LARS sense amino acid levels and thereby regulate mTOR signaling pathway [97] (Fig. 2a). The Rag GTPase promotes the localization of mTORC1 to the lysosomal surface and activation. Rag GTPase is further regulated by amino acids through sensors-GATOR2-GATOR1 axis. GATOR1, a negative regulator of mTORC1, interacts with Rag, leading to the inhibition of mTORC1 activity, while GATOR2 modulates mTORC1 activity by inhibiting GATOR1. Sestrins and SAR1B are leucine sensors in cytosolic. In situations of leucine deprivation, they bind to and inhibit GATOR2, a positive regulator of mTORC1 [98, 99]. Leucine can bind to sestrins and SAR1B, dissociating GATOR2 from the complex to activate mTORC1. Furthermore, in cases of amino acid deficiency, the general control nonderepressible 2 (GCN2)/ATF4 pathway is activated by uncharged tRNA, leading to the upregulation of sestrins expression to inhibit mTORC1 activity. Similar to sestrins, in arginine-depleted conditions, CASTOR1/2 form either a CASTOR1 homodimer or CASTOR1/2 heterodimer to inhibit GATOR2 and subsequently inhibit mTORC1 activity [100]. Arginine disrupts the CASTOR1-GATOR2 complex by binding to CASTOR1, and activates GATOR2 to stimulate mTORC1. SAMTOR senses the changes of intracellular methionine concentration in the form of SAM. SAM disrupts the SAMTOR-GATOR1 complex by binding directly to SAMTOR, and reduces the GTPase-activating protein (GAP) activity of GATOR1, which then activates the mTORC1 signaling pathway [101]. With adequate amino acids, the E3 ubiquitin ligase KLHL22 acts as a positive regulator of mTORC1 by promoting the degradation of GATOR1 [102]. Leucyl-tRNA synthetase (LARS) senses intracellular leucine and directly activates mTORC1 activation by directly interacting with Rag rather than acting with GATOR1/2 [103]. LARS mediates the leucylation of RagA/B, which subsequently activates mTORC1 [103]. Consistently, abovementioned amino acid sensors eventually active Rag, and mediate lysosomal translocation of mTORC1 [104], a critical step in the activation of the mTORC1. When mTORC1 is activated at lysosomal membrane, autophagy is inhibited and tumorigenesis is promoted.

In addition to above cytoplasm sensors, lysosomal sensors also play a crucial role in mTORC1 activation. Ragulator complex provides a platform for lysosome to tether Rag. The Ragulator interacts with the Rag heterodimers in an amino acid- and v-ATPase-dependent fashion, which finally activates mTORC1 [105]. SLC38A9, a lysosomal membrane protein with homology to amino acid transporters, participating in the activation of mTORC1 signaling pathway by influencing Rag [106]. Mechanically, SLC38A9 stimulates the release of GDP from Rag A upon activation by arginine. This action propels Rag into the activated state, subsequently activating mTORC1 [107]. Notably, SLC38A9 is crucial for the efflux of leucine, glutamine, tyrosine, and phenylalanine generated from lysosomal proteolysis. This efflux is necessary to activate mTORC1 through cytoplasmic sensors [108]. Thus, lysosomal sensors allow for the integration of lysosomal nutrient information into the regulation of mTORC1 activity. Collectively, amino acids are not only sources for energy and protein synthesis in tumorigenesis, but also act on mTORC1 as signaling molecules.

Meanwhile, mTORC1 can also regulate amino acid metabolism through its downstream signaling effectors (Fig. 2a). In response to growth signals, mTORC1 activates ATF4 to stimulate enzymes in serine synthesis for folate cycle and purine biosynthesis [109]. Besides, signaling effector ATF4 also transcriptionally regulates serine transporters SLC1A5 and therefore facilitates the uptake of serine [110]. Astutely, ATF4 can also regulate the expression of other amino acid transporters such as CHAC1, SESN2, SLC7A11, SLC7A5, SLC7A1, and SLC3A2, and increase amino acid uptake [111]. Upon the accumulation of glutamine, mTORC1 downregulates miR-23a and miR-23b and subsequently promotes GLS expression to accelerate glutamine catabolism. When glutamine is deficient, mTORC1 represses the transcription of GDH inhibitor SIRT4, prompting glutamine anaplerosis [112]. mTORC1 also activates arginine catabolism by promoting ODC expression in RAS transformed cells to promote polyamine production and tumor progression. Mechanically, mTORC1 promotes the association between ODC mRNA and mRNA-binding protein, promoting ODC mRNA stabilization and expression [113]. Besides, positively regulated mTORC1 leads to the stabilization of MYC, which in turn induces ASS1 expression by competing with HIF1α for ASS1 promoter binding sites and therefore promotes arginine expression [114]. Collectively, mTORC1 regulates amino acid metabolism through multiple signaling effectors, including amino acid transporters, synthetic and catabolic enzymes. mTORC1 downstream signaling molecule MYC also plays extensive regulatory roles in amino acid metabolism.

### *Myc* drives amino acid metabolism

*Myc* is a proto-oncogene which encodes transcription factor MYC, constitutively expressed in tumor and associated with altered metabolism [115]. MYC directly regulates key metabolic enzymes expression, resulting in altered metabolism like increased nutrient uptake, enhanced glycolysis, and elevated fatty acid and nucleotide synthesis [116]. Amino acid metabolism, both EAAs and NEAAs are also regulated by MYC (Fig. 2b).

As EAAs rely on external resources, corresponding amino acid transporters are crucial and often up regulated in cancer. A positive feedback circuit called MYC-SLC7A5/SLC43A1 is critical in EAAs metabolism in tumor. SLC7A5 imports EAAs in exchange for glutamine export, while SLC43A1 facilitates the import of large neutral essential amino acids (LNEAAs) like BCAAs and tryptophan [117, 118]. MYC plays a pivotal role in promoting the transcription of SLC7A5/SLC43A1 and consequently the uptake of EAAs, which in turn activates mTORC1 and accelerates *Myc* transcription. When SLC7A5/SLC43A1 is blocked and thus amino acids uptake is decreased, the GCN2-eIF2α amino acid stress response pathway will be triggered, leading to the inhibition of MYC mRNA translation. Collectively, this circuit leads to a cascade that affects the entire amino acid metabolic process and oncogene transcription, ultimately promoting tumorigenesis [119]. Specifically, MYC can enhance tryptophan uptake by upregulating the expression of transporters such as SLC7A5 and SLC1A5. Additionally, it can promote the conversion of tryptophan to kynurenine by inducing arylformamidase (AFMID) within the kynurenine pathway [120, 121]. Elevated level of kynurenine has been found to help tumors to evade immune surveillance [122]. Additionally, *Myc* upregulates BCAT1, a crucial enzyme in BCAAs catabolism, and increases biosynthesis and promotes tumor development [123].

In addition to above regulation of EAAs, *Myc* can also catalyze NEAAs metabolism, like glutamine, proline, and serine. Besides glucose, glutamine could function as major fuels in tumors. MYC prompts the expression of glutamine transporters such as SLC1A5 and SLC38A5 [122]. In addition, *Myc* also plays a part in glutamine anabolism and catabolism. MYC can demethylate the promoter of GS, prompting the synthesis of glutamine in cancer cells [124]. However, *Myc* also participates in glutamine catabolism through acting on miR-23a/b in some cancers. miR-23a/b can suppress the expression of GLS and then facilitate glutaminolysis in tumor cells [125]. Collectively, MYC functions differently in different types of tumor cells, and metabolic requirements differential within specific cancer types might dictate the outcome of glutamine metabolism regulated by MYC.

NEAA proline can be synthesized by aldehyde dehydrogenase family 18 member A1 (ALDH18A1, P5CS) and pyrroline-5-carboxylate reductase (PYCR) from glutamine and arginine, thus MYC induced P5CS and PYCR upregulation can promote the proliferation and invasion of cancer cells [28, 123]. In this way, the biosynthesis of glutamine-to-proline is prompted, assisting tumor cells to alleviate ER stress and promote proline homeostasis. MYC increases miR-23b to decrease the expression of proline oxidase/proline dehydrogenase (POX/PRODH), leading to the inhibition of proline catabolism [126]. Thus, MYC can not only promote the synthesis of proline, but also inhibit its break down [127].

MYC also participates in serine metabolism. Enhanced activity of MYC activates metabolic enzymes in SSP such as PHGDH, PSAT and PSPH, resulting in enhanced serine production [123]. MYC upregulates SHMT2, which is critical for maintaining cellular redox homeostasis. Under the catalyze of SHMT2, serine metabolized glycine directly participates in glutathione (GSH) production. In this way, MYC promotes GSH synthesis de novo, and then finally resists oxidant to promote tumor progression. MYC upregulates SHMT2, leading to increased production of one-carbon unit 5’m-THF and therefore the generation of NADPH. NADPH also plays a crucial role in maintaining the redox balance by reducing GSSG to GSH [128]. Collectively, MYC promotes serine synthesis and thereby GSH and NADPH production to resist oxidation and promote tumor growth.

### Altered KRAS and amino acid metabolism

KRAS, a frequently mutated oncogenic protein in human cancers, plays a pivotal role in regulating MYC and mTOR activity through RAF-MEK-ERK and PI3K-AKT pathways, respectively [129, 130] (Fig. 2c). The mutation impairs its GTPase activity, leading to persistent activation of downstream signaling cascades, influencing the cellular metabolism and promoting tumor cell proliferation. Amino acid metabolism is also regulated by KRAS, and targeting the metabolic network downstream of KRAS may offer potential avenues for treating KRAS-driven tumors [131].

KRAS-induced macropinocytosis maintains intracellular glutamine levels. Besides, KRAS regulates enzymes in glutamine catabolism. KRAS downregulates glutamate dehydrogenase1(GLUD1), leading to alterations in glutamine metabolism to produce NEAAs instead of participating in TCA cycle. Besides, KRAS upregulates GOT2 in the mitochondrial and GOT1 in the cytoplasm [132]. Under such circumstance, glutamine-derived aspartate is converted into OAA by GOT1 in the cytoplasm, and finally converted into pyruvate, resulting in the production of NADPH to maintain the cellular redox balance. Thus, *Kras*-mutated cells resist cisplatin treatment by upregulating glutamine consumption to maintain a redox state. As upregulated glutamine catabolism is essential for tumor but dispensable for normal cells, inhibiting enzymes like GOT1 in the glutamine catabolic pathway leads to increased levels of reactive oxygen species (ROS), reducing the levels of GSH and ultimately inhibiting tumor growth [133].

## Amino acids in tumor microenvironment

Tumors thrive within the intricate TME, which is complex and continuously evolving including surrounding blood vessels, immune cells, fibroblasts and the extracellular matrix (ECM) [134]. The bidirectional interaction between tumor and TME takes various forms. Tumors assimilate essential nutrients through macropinocytosis to satisfy its vigorous metabolism. Thus, rapidly proliferating tumor cells compete for relatively scarce nutrients with fibroblasts and immune cells, shaping a commonly hypoxic, acidic, and nutrient-deprived TME. Collectively, the TME promotes tumor progression and immune evasion through nutrients deprivation. Besides, tumors secrete various bioactive molecules that profoundly influence the TME.

### Macropinocytosis in TME takes up amino acids

**Fig. 3:**
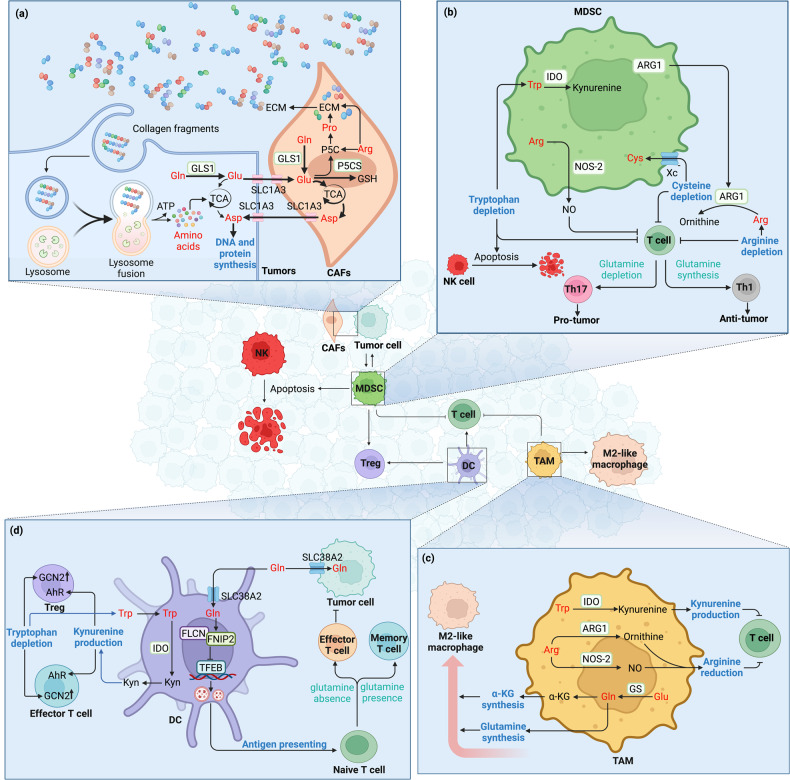
Metabolic relationship between immune cells and cancer cells in TME. **a** Tumor macropinocytosis and tumor interaction with CAFs. Collagen fragments secreted by CAFs are presented in the TME, and are taken up by tumor cells through macropinocytosis and catabolized to support tumor cells survival. CAFs supply aspartate to cancer cells to synthesis DNA and protein via SLC1A3 while cancer cells in turn secrete glutamine-derived glutamate to CAFs via SLC1A3 to generate GSH and therefore maintain the redox state. In CAFs, both glutamine and arginine facilitates proline synthesis and ECM secretion. **b** Reprogramed amino acid metabolism in MDSCs inhibits T cell and NK cell. MDSCs highly express ARG1 to deprive arginine in TME, impairing T cell-mediated anti-tumor immunity. MDSCs express x_c_^-^ transporter and import cystine, causing depleted cysteine and impaired T cell function. Overexpression of IDO in MDSCs deprives tryptophan, leading to T cell stagnating in G0 and NK cell apoptosis. The differentiation of naive T cells into Th1 or Th17 cells depends on glutaminolysis. This is because lacked glutamine promotes Th1 cells while inhibits Th17 cells. MDSCs also highly express NOS-2, producing NO to impair anti-tumor effect of T cells. **c** Reprogramed amino acid metabolism in TAMs polarizes macrophage and suppresses T cell. The expression of GS in TAMs increased, promoting glutamine synthesis and M2-like macrophage polarization. TAMs also overexpress IDO to produce kynurenine, blocking T cell proliferation and inducing T cell death. ARG1 overexpression depletes arginine and thereby suppresses T cell function. It is worth noting that NOS-2 is also upregulated to produce NO and suppress T cell function. **d** Reprogrammed glutamine and tryptophan metabolism in DC influences T cell. Upregulated IDO expression in DC induces kynurenine production and tryptophan depletion as well as the activation of AhR and GCN2 respectively, leading to CD8^+^ T cell dysfunction and Treg differentiation. DC and tumor cells both express SLC38A2 to mediate glutamine uptake to tune anti-tumor immunity. Glutamine in DC promotes the formation of the FLCN-FNIP2 complex and thereby restricts TFEB activity, promoting antigen cross-presentation and T cell immunity. Glutaminolysis underlies asymmetric T cell division. The daughter T cell proximal to the APC is accumulated with glutamine transporters and adopts an effector-like fate, while the distal T cell absence of glutamine transporters assumes a memory-like fate. CAFs Cancer-associated fibroblasts, ECM Extracellular matrix, MDSC Myeloid-derived suppressor cell, TAM Tumor-associated macrophage, GCN2 General control nonderepressible2, AhR Aryl hydrocarbon receptor, Kyn Kynurenine, DC Dendritic cell, NK Natural killer cell, Cys Cysteine.

Macropinocytosis is a type of endocytosis that involves the nonspecific uptake of extracellular nutrient molecules like proteins and amino acids [135] (Fig. 3). Even targeted drugs block enzymes in vital biosynthesis process, cancer cells still take up necessary biological materials from TME (e.g., collagen fragments) through macropinocytosis to maintain proliferation. Macropinosomes formation is an actin-dependent process that is initiated upon stimulation of growth factors like colony stimulating factor (CSF-1), epidermal growth factor (EGF), or platelet-derived growth factor. Besides, oncogenic mutations in *Kras* or PI3K pathway activation can also drive micropinocytosis [136]. Proteins ingested through macropinocytosis can be decomposed into free amino acids by cellular autophagy for new protein synthesis, or catabolized to generate ATP for energy supply [137]. Therefore, macropinocytosis enables tumor cells to survive in harsh environment by providing both materials and energy.

Macropinocytosis is a metabolic adaptation to nutrient stress, thus amino acids depletion drives macropinocytosis in cancers to obtain nutrients. Mechanically, a low amino acid environment inhibits the Hippo pathway and promotes membrane localization of EGFR and TGFBRII, triggering macropinocytosis [138]. When intracellular free amino acids are abundant, mTORC1 precisely controls the utilization of extracellular protein-derived amino acids within lysosomes by repressing macropinocytosed protein catabolism [139].

### Amino acid metabolism and CAFs

TME is hypoxia and devoid of nutrients, which is unfavorable for the survival of cancer cells. Therefore, cancer cells cleverly hold fibroblasts in TME, converting them into CAFs to create an altered homeostasis suitable for tumor growth [138]. CAFs enhance tumor invasion through their bidirectional interaction with tumor cells and highly secretory phenotype to produce ECM [140] (Fig. 3). CAFs regulate amino acid metabolism to promote multiple processes like tumorigenesis, angiogenesis, and metastasis [141]. CAFs functional significance in cancer makes them attractive targets for cancer treatment.

CAFs play a critical role in tumor progression by interacting with tumor cells and modulating tumor metabolism through amino acid transport (Fig. 3). In the stiff matrix of TME, ECM mechanotransduction re-localizes YAP/TAZ to the nucleus and activates the transcription of GLS1 and SLC1A3 in both cancer cells and CAFs [142, 143]. GLS1 promotes the transformation from glutamine to glutamate. However, upregulated-glutamate does not contribute to the TCA cycle in tumors, while in CAFs, glutamate is a major source of carbon for the TCA cycle and produces asparate. SLC1A3 is upregulated simultaneously in tumor cells and CAFs, but functions differently. SLC1A3 in CAFs could provide aspartate to cancer cells, while cancer cells in turn secrete glutamine-derived glutamate through SLC1A3 to CAFs. In such metabolic crosstalk between CAFs and cancer cells, CAF-derived aspartate promotes pyrimidine and protein synthesis to sustains cancer cells proliferation, while cancer cell-derived glutamate balances the redox state of CAFs to promote ECM remodeling. Drugs targeting SLC1A3 can significantly reduce tumor growth due to the close interaction and extensive metabolic remodeling between CAFs and tumors. The amino acid metabolism interaction between CAFs and cancer cells have been recently reviewed in detail [141, 144].

CAFs are known to be the most important cells for producing ECM. Collagen, rich in proline, is the main component of ECM, the degradation of which could provide materials and energy to tumor cells. Proline synthesis is sustained through the conversion of glutamate catalyzed by P5C synthase (P5CS) or arginine into pyrroline-5-carboxylate (P5C). P5C serves as the ultimate precursor for proline and, consequently, collagen synthesis. Proline synthesis is upregulated in CAFs and acts as a limiting factor in ECM production. Thus, P5CS deletion decreases collagen and therefore ECM production, which could be rescued with proline supplementation [145].

### Amino acid metabolism and immune cells

TME comprises diverse cell types, including immunosuppressive cells like myeloid-derived suppressor cells (MDSCs), TAMs, and regulatory T cells (Tregs), as well as tumor-antagonizing immune cells like natural killer cells (NKs), T lymphocytes, B lymphocytes and dendritic cells (DCs). Although the tumor-antagonizing immune cells within TME tend to target and kill the cancer cells in the early stage of tumorigenesis, cancer cells eventually escape immune surveillance through various mechanisms, including reprogramming of metabolism.

Tumor associated MDSCs highly express ARG1 (Fig. 3), depriving arginine in TME, leading to the lack of arginine in T cells and impairing T cell-mediated anti-tumor immunity [146]. Besides, MDSCs also highly express NOS-2, not only decompose arginine but also produce NO to impair anti-tumor effect of T cells [147]. MDSCs express the x_c_^-^ transporter to import cystine. Thus, in the presence of MDSCs, cysteine is reduced and T cell function is impaired [148]. Apart from depleting arginine and cysteine, MDSCs also inhibit T cell function by expressing IDO. In tumor site and drainage lymph nodes, IDO overexpression in MDSCs deprived tryptophan in TME, which is necessary for T cell and NK cell proliferation. This eventually leads to T cell stagnating in G0 and NK cell apoptosis [149]. IDO also generates kynurenine, which has been reported to block T cell proliferation and even induce T cell death as mentioned above. TAM also creates an immunosuppressive TME, which tightly links to glutamine metabolism [150]. In nutrient-deprived tumor stroma, GS expression in TAM increased to promote glutamine synthesis. Increased GS provides metabolic condition skews macrophages toward an M2-like, pro-metastatic macrophages by providing more glutamine and α-KG [151, 152]. Similar to MDSC, TAM also overexpresses IDO and ARG1 to deplete tryptophan and arginine as well as produce kynurenine to suppress T cell function [153]. It is worth noting that NOS-2 is also upregulated in TAM to reduce arginine and suppress T cell function.

Tumor-antagonizing immune cells function is also suppressed in the TME due to altered amino acid metabolism, thereby influencing the development of tumors. During T cell activation and differentiation, amino acids play dual roles as both energy source and substrates for protein and nucleic acid biosynthesis [154]. Compared to naive CD8^+^ T cells, activated CD8^+^ T cells increases the density of SLC1A5 and SLC7A5 on the cell surface to enhance glutamine uptake. Glutaminolysis underlies asymmetric T cell division, as glutaminolysis and mTORC1 activation is necessary to maintain c-Myc asymmetry. Asymmetric c-Myc levels in daughter T cells would affect their proliferation, metabolism, and differentiation [155]. Thus, the daughter T cell proximal to the antigen presenting cell is accumulated with glutamine transporters and adopts an effector-like fate, while the distal T cell absence of glutamine transporters assumes a memory-like fate. The differentiation of naive T cells into Th1 or Th17 cells also depends on glutaminolysis. When GLS is inhibited, the number of Th1 cells increases while the number of Th17 cells decreases [156]. Besides, glutamine limitation also promotes Treg differentiation [157]. IDO is activated in DCs to deplete tryptophan and produce kynurenine. As previously mentioned, kinase GCN2 is activated by elevated levels of uncharged tryptophan tRNA, triggering CD8^+^ T cell-cycle arrest and functional anergy [158]. Moreover, GCN2 activation fosters de novo Treg differentiation and enhances suppressor function in mature Tregs [159]. On other hand, kynurenine overexpression not only impairs effector T cell, but also promotes the differentiation of Treg through the activation of AhR [160]. DCs and tumor cells both express SLC38A2 to facilitate glutamine uptake, modulating anti-tumor immunity. Mechanistically, glutamine acts as an intercellular metabolic checkpoint that licenses DCs function in activating CD8^+^ T cells [161]. Glutamine promotes the formation of the FLCN-FNIP2 complex, consequently restricting TFEB activity. TFEB acts as a molecular switch, regulating exogenous antigen-presentation pathways through lysosome activation and thus influencing CD8^+^ T cell activity.

As mentioned above, arginine metabolism regulates immune response and responsible for tumor progression [162]. Arginine metabolic enzyme NOS-2 directs the polarization of gamma delta T cells towards a pro-tumoral phenotype, thereby inducing metastatic progression [163]. Asparagine up regulates the LCK signaling pathway to enhance CD8^+^ T cell activity and thereby inhibit tumor growth. This subverts the previous understanding of the cancer-promoting function of asparagine [164]. Collectively, above findings reveal that amino acid metabolism in tumors and immune cells plays vital roles in the process of tumorigenesis and development.

## Amino acid metabolism and epigenetic modification

Epigenetics refers to heritable phenotype changes that do not involve alterations in the DNA sequence [165]. It encompasses various mechanisms, including DNA methylation, histone modifications, chromatin remodeling, and small RNA regulation. Amino acid metabolites like SAM and acetyl-CoA are essential substrates for epigenetic modification, while amino acid metabolism also requires epigentic modification of associated metabolic enzymes [166]. This reciprocal regulatory relationship has a profound impact on tumor progression.

### Methylation

**Fig. 4:**
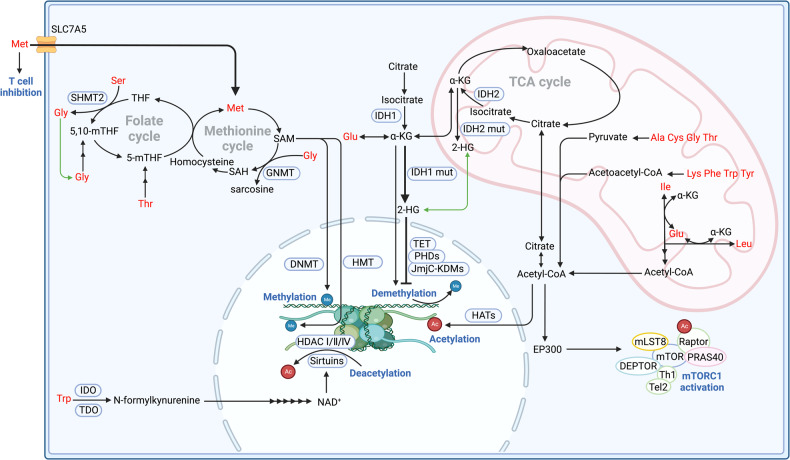
Amino acid metabolism and epigenetic modification. Tumors often overexpress SLC7A5 to obtain methionine, competing with T cell to inhibit T cell function as well as participating in methionine cycle to synthesis SAM. The downregulation of glycine or the knock down of GNMTs in the methionine cycle reduces the consumption of SAM and thus promotes the methylation of histone by HMT and DNA by DNMT. Serine fuels folate cycle under the catalyze of SHMT2 and produces glycine. Glycine can also be metabolized to produce 5,10-mTHF to fuel the folate cycle. Threonine depletion from the culture medium decreases folate cycle and the levels of SAM as it serves as the precursor for 5-mTHF synthesis. Mutated IDH, in contrast with common IDH, generates 2-HG which suppresses DNA demethylation by KDMs, leading to a hypermethylation phenotype. Isoleucine and leucine catabolism directly generates acetyl-CoA in the mitochondria. Alanine, cystine, glycine and threonine convert to pyruvate to synthesis acetyl-CoA. Lysine, phenylalanine, tryptophan and tyrosine convert to acetoacetyl-CoA to synthesis acetyl-CoA. Collectively, above amino acids derived acetyl-CoA donates acetyl groups to modify histone proteins, which is catalyzed by HATs. An alternative pathway for isoleucine and leucine to activate mTORC1 involves providing acetyl-CoA to the EP300 acetyltransferase. This enzyme mediates inhibitory acetylation of the mTORC1 regulator Raptor, ultimately leading to mTORC1 activation. Deacetylation refers to acetyl groups remove from histones catalyzed by HDAC. The sirtuin proteins are classified as class III HDACs due to their dependence on NAD^+^ for catalysis, in contrast to the zinc-dependent catalysis observed in class I, II, and IV enzymes. The tryptophan metabolism and NAD^+^/NADH ratio exhibits a close correlation with both the acetylation state and energy status. SAH S-Adenosyl-homocysteine, GNMTs glycine N-methyltransferase, DNMTs DNA methyltransferases, HMT Histone methyltransferase, 2-HG 2-hydroxyglutaric acid, IDH Isocitrate dehydrogenase, HATs Histone acetyltransferases, Thr Threonine, Ile Isoleucine.

SAM is synthesized from methionine and functions as the primary methyl donor in various methylation reactions, mainly DNA methylation (Fig. 4). DNA methylation is catalyzed by DNA methyltransferases, which transfer methyl group from SAM to DNA. As a methionine transporter, SLC7A5 is crucial for intracellular methionine concentration. Disruption in methionine concentration is closely associated with cancer development, as DNA hypermethylation usually suppresses tumor suppressor genes expression [167].

Tumorigenesis is also affected when methylation occurs on histones [168]. The effect of histone methylation depends on which amino acid residue is methylated and how many methyl groups are involved [10]. Specifically, arginine methylation on histone can promote transcriptional activation, while lysine methylation on histone involves both transcriptional activation and repression [169]. When it terms to transcriptional repression, tumors always overexpress methionine transporter SLC43A2 to outcompete T cells for methionine. Methionine deficiency in T cells leads to downregulation of H3K79me2, promoting CD8^+^ T cell death and inhibiting Th17 polarization [170]. When it terms to transcriptional activation, downregulation of SLC7A5 in T cells restricts methionine absorption, and alters H3K27me3 deposition at the promoters of key T cell stemness genes. These changes promote the maintenance of a ‘stem-like memory’ state and improve long-term persistence and anti-tumor efficacy [171]. In conclusion, methionine metabolism regulates genomic architecture, chromatin dynamics and gene expression by dynamically modulating methylation on DNA and histone [172].

In addition to methionine, other amino acids can also indirectly regulate the level of methylation. Threonine metabolizes into 5-mTHF while serine metabolizes into 5,10-mTHF, playing pivotal roles in cell fate by contributing to SAM synthesis and thereby methylation status. Previous studies reveal that threonine depletion decreased the levels of SAM, leading to decreased tumor growth and increased differentiation [173]. Transaminase like glycine N-methyltransferase (GNMT) catalyzes the transfer of a methyl group from SAM to glycine to form sarcosine, leading to SAM depletion and S-Adenosyl-homocysteine (SAH) accumulation [174]. The ratio of SAM/SAH affects methylation [175]. Therefore, knock down of transaminase like GNMT or the dysregulation of glycine clearly affects the methylation of histone and DNA, affecting gene expression and tumorigenesis [176, 177]. Moreover, glycine can also be metabolized to produce 5,10-mTHF to fuel folate cycle, and thereby regulating methionine cycle.

### Demethylation

Amino acid like glutamine can be catabolized to produce α-KG. Meanwhile, through α-KG-dependent dioxygenase, the methylation of histone and DNA can be removed [178]. The family of α-KG-dependent dioxygenases includes Jumonji C-domain lysine demethylases (JmjC-KDMs), ten-eleven translocation (TET) DNA cytosine-oxidizing enzymes, and prolyl hydroxylases (PHDs) [179].

Normal isocitrate dehydrogenases (cytoplasmic, IDH1; mitochondrial IDH2) catalyze isocitrate to produce α-KG in TCA cycle to fuel tumors. However, IDH is the most frequently mutated metabolic genes in human cancer. When IDH is mutated, it can further catalyze α-KG to 2-hydroxyglutaric acid (2-HG), and 2-HG can competitively inhibit α-KG-dependent dioxygenases [180]. Thus, mutated IDH inhibits DNA and histone demethylation, leading to a hypermethylation phenotype and promoting the differentiation of cancer stem cells [181]. In IDH mutant glioblastoma, the CpG island in promoter region of BCAT1 is usually hypermethylated. The inhibition of BCAT1 expression results in the reduction of glutamate and therefore inhibit tumor growth and invasion [182]. Consistently, individuals diagnosed with IDH^WT^/TET2^WT^ myeloid leukemia, α-KG is maintained at normal levels and therefore BCAT1 expression is promoted compared to IDH^mut^/TET^mut^ myeloid leukemia. Elevated expression of BCAT1 serves as a robust indicator for poorer survival prognosis, which level has been observed significantly escalates upon disease relapse [183].

### Acetylation and deacetylation

Histone acetylation is a process in which acetyl-CoA donates acetyl groups to modify histone proteins catalyzed by histone acetyltransferases (HATs) [184]. Both acetyl-CoA abundance and the ratio of acetyl-CoA to coenzyme A regulate histone acetylation in cancer [185]. Histone acetylation leads to attractive force reduction between histones and DNA, promoting a more open chromatin structure that allows for increased transcriptional activity [11, 186]. Furthermore, acetyl-CoA derived from amino acid metabolism also plays a role in non-histone protein acetylation and regulates tumor growth.

Acetyl-CoA level and amino acid metabolism affects each other. BCAAs like isoleucine and leucine synthesize acetyl-CoA under the catalyze of transaminase. Other amino acids such as lysine, phenylalanine, tryptophan, and tyrosine also contribute to acetyl-CoA production by forming acetoacetyl-CoA. Similarly, alanine, serine, tryptophan, cysteine, glycine, and threonine synthesize acetyl-CoA through pyruvate formation. Leucine metabolism provides acetyl-CoA to the EP300 acetyltransferase, leading to acetylation of mTORC1 regulator Raptor. This acetylation ultimately results in mTORC1 activation and altered amino acid metabolism [187]. Isoleucine and leucine catabolism generates acetyl-CoA within the mitochondria. Subsequently, mitochondrial acetyl-CoA must be transported to the cytoplasm and nucleus to regulate gene expression through epigenetic mechanisms [188].

Deacetylation refers to the removal of acetyl groups from histones, which is typically catalyzed by histone deacetylase (HDAC). Histone deacetylation results in a more condensed form of DNA known as heterochromatin, which is linked to reduced levels of gene transcription. The sirtuin proteins are classified as the class III HDACs depending on NAD^+^ in contrast to the zinc-dependent catalysis by Class I, II, and IV enzymes [189]. Thus, the tryptophan metabolism and therefore NAD^+^/NADH ratio exhibit a close correlation with both the acetylation state and energy status, playing important roles in inhibiting gene expression and thereby influencing tumor progression.

### Phosphorylation, succinylation, and lactylation

In addition to the aforementioned types of epigenetic modifications, it is noteworthy to consider other post-translational modifications (PTMs), including phosphorylation, succinylation, and lactylation. Each of these PTMs alters the charge and structure of proteins. For instance, modifications to histones affect their binding to DNA, thereby influencing chromatin status and gene expression [190]. PTMs of enzymes influence their binding to substrates, thereby affecting various metabolic processes.

Serine, threonine as well as tyrosine residues have established as residues for histone phosphorylation [191]. AMP-activated protein kinase (AMPK) is a sensor of cellular energy status and phosphorylates a variety of cellular substrates include histones [192]. As a nutrient sensor, AMPK can be activated under a variety of stress conditions. Recent studies indicate that AMPK is activated by the amino acids like alanine [193], aspartate [194] and cysteine [194] and then phosphorylates histones. Histone phosphorylation is linked to various cellular processes, including transcriptional activation, mitosis, DNA repair, and apoptosis [195]. Phosphorylation also occurs in enzymes related to amino acid metabolism. When phosphorylation occurs in branched chain ketoacid dehydrogenase kinase, it regulates EMT genes and leads to the metastasis of colorectal cancer [196]. Besides, phosphorylation of GLS is essential for its enzymatic activity and critically contributes to tumorigenesis [197].

Succinylation is an innovative PTM where a succinyl group is added to a lysine residue. This modification is associated with the catabolism of BCAAs, as the generation of succinyl-CoA serves as an intermediate for succinylation [198]. The transcriptional characteristics of succinylated histones resemble those observed in acetylated counterparts, thereby activating gene expression [198, 199]. Therefore, succinyl-CoA accumulation may enhance cancer initiation and progression by promoting a global succinylation program that favors cancer growth [200]. Enzymes involved in amino acid metabolism can also be succinylated. Upon oxidative stress, enhanced succinylation of GLS leads to increased oligomerization and activity, thereby promoting glutaminolysis and tumor growth [201].

Lactylation is also a novel epigenetic modification, in which lactic acid modifies the lysine residues [202]. Hypoxia is linked to elevated lactate levels originating from heightened glycolysis activity, thereby enhancing intracellular histone lactylation [203]. Histone lactylation can affect gene expression in tumors and immunological cells, thereby promoting malignancy and immunosuppression. In tumor-infiltrating myeloid cells, a potent accumulation of lactate occurs, leading to the upregulation of methyltransferase-like 3 (METTL3) through lactylation. The increased expression of METTL3 in these cells was correlated with the poor prognosis of patients [204].

## Targeting amino acid metabolism in tumor therapy

Primary strategies in cancer treatment involve targeting disparities between normal and tumor cells in gene expression and phenotype. Metabolism alteration in tumors is a noteworthy phenotype. Amino acids metabolism, being fundamental to vital biological processes, play a crucial role in tumor initiation and progression [5]. Consequently, targeting amino acid metabolism like amino acid depletion is significant in cancer treatment approach [7]. Strategies for targeting amino acid metabolism encompass inhibiting amino acid transporters, regulating amino acid biosynthesis and consumption as well as developing amino acids modified dietary (Table 1). Given different metabolism in different tumors, developing tailored strategy for distinct tumors becomes imperative.

**Table 1:** Key enzymes/transporters and their drugs in cancer amino acid metabolism.

	Target	Drug	Cancer type	Clinical phases
Amino acid transporters	ASCT2	Tamoxifen and Raloxifene	Breast cancer	Approved
	ASCT2	PGS-siRNA	NSCLC	Preclinical
	ASCT2	V-9302	HCC	Preclinical
	CATs	verapamil	Colorectal cancer	Preclinical
	SLC6A14	Aspartate-modified docetaxel-loading stealth liposomes	Lung cancer	Preclinical
Asparagine metabolism	ASNS	metformin	Multiple cancers	Preclinical
		Asparlas	Acute lymphoblastic leukemia	Approved
Arginine metabolism	ADI	ADI-PEG20	Ovarian cancer	Phase III
		INCB001158	Advanced or metastatic solid tumors	Phase II
Glutamine metabolism	GLS	Telaglenastat	NSCLC, lymphoma, glioma, breast cancer, pancreatic cancer, and kidney cancer [234]	Phase II
	GLS1	968	NSCLC [235]	Preclinical
One-carbon metabolism	DHFR	Methotrexate	Multiple cancers	Approved
	Thymidylate synthase	5-fluorouracil	Multiple cancers	Approved

### Amino acid transporters inhibition

Amino acids substantial uptake occurs in tumor cells which overexpress amino acid transporters. Consequently, targeting these transporters to restrict amino acid availability is an effective strategy for inhibiting tumor growth. Drugs targeting amino acid transporters and related enzymes have transitioned from preclinical research to clinical trials and have demonstrated efficacy in some cases [205]. Glutamine transporters usually highly expressed and associated with poor prognosis as external glutamine is essential for cancer cells to survive [206, 207]. This differentiates cancer cells from normal cells, providing a target for tumor therapy. Clinically, Tamoxifen and Raloxifene block glutamine uptake by inhibiting ASCT2 expression in breast cancer to suppress tumor [208]. Likewise, pharmacological blockade of ASCT2 with V-9302 also resultes in attenuated cancer cell growth and proliferation [209]. However, therapy targeting ASCT2 alone is not enough, as there are compensation from other transporters like cystine/glutamate antiporter(xCT) to replace ASCT2 to fuel tumors. Cationic amino acid transporters like CAT-1, CAT-2 and CAT-3 for lysine, arginine, and histidine are also dysregulated in tumors and associated with drug resistance. Specifically, CAT-1 expression exhibits a correlation with tumor grade in prostate cancer [208]. It also plays a pivotal role in promoting growth, proliferation, and metastasis of colorectal cancer and breast cancer [210]. Upregulated CAT-3 increases arginine uptake and thereby induces tumors to adapt glutamine deprivation [39]. Downregulation of CATs (CAT-1, CAT-3) through lentiviral transduction with shRNAs or chemical like verapamil shuts down tumor proliferation and induces death [211, 212]. Conversely, loss of CAT-2 exacerbates inflammation-associated colon tumorigenesis [213].

Apart from targeting these tranporters directly to prevent amino acids uptake, these tranporters can also be targeted to deliver drugs. The glutamine macromolecular analog polyglutamine (PGS) can mimic glutamine and selectively ferry siRNA through ASCT2. Therefore, siRNA delivered by PGS targets the growth and survival of certain cancer [214]. Thus, RNAi in combination with chemotherapy can augment the anti-tumor effect. Apart from ASCT2, SLC6A14 is another amino acid transporter that demonstrates upregulation in numerous cancer types [215]. Hence, SLC6A14 emerges as a promising target for tumor therapy, with its potential extending to enabling the selective delivery of anticancer drugs to tumor cells. Stealth liposomal systems functionalized with the aspartate-polyoxyethylene stearate conjugate (APS) are developed for transporter-mediated targeted delivery of docetaxel to SLC6A14, resulting in a significantly efficiency for delivering anticancer drugs into cells [216].

### Amino acid metabolism enzymatic inhibition

Amino acid metabolism is also reglulated by biosynthetic enzyme and catabolic enzyme. Numerous pharmacological inhibitors targeting key enzymes in amino acid metabolism undergo extensive research. Asparagine is the most successful and well-documented target in amino acid depletion therapy, especially in acute lymphoblastic leukemia [217]. Asparlas, a drug frequently used in clinical practice, is developed for the treatment of acute lymphoblastic leukemia. It acts as a long-acting asparagine-specific enzyme to deplete asparagine [218]. Studies shown that inhibited ASNS or metformin inhibited ETC limits tumor asparagine synthesis, impairing tumor growth in multiple mouse models [219]. *Kras* mutation activates the ATF4 signaling pathway through its downstream AKT and NRF2 in NSCLC. When ASNS is inhibited by AKT and the extracellular asparagine is depleted simultaneously, tumor growth can be reduced [220]. Therefore, ASNS is also a promising therapeutic target for *Kras*-mutated NSCLC.

Arginine is another targeted amino acid in depletion therapy. As mentioned above, ASS1, a rate-limiting enzyme for synthesizing arginine, is frequently deficient in tumors and thereby arginine is depleted. Meanwhile, enzymes such as arginine deiminase (ADI) and ARG I could also exhaust extracellular arginine by transforming arginine into citrulline and ornithine, respectively. Thus, depleting arginine by ADI-PEG20 or rhArg1-PEG to downregulate arginine level in serum is essential to restrain cancer cells proliferation [221, 222]. Since ARG is consistently overexpressed in both cancer cells and MDSC, the ARG inhibitor INCB001158 has the potential to normalize arginine levels within TME, consequently revitalizing T cell functionality [223]. Simultaneously, the intracellular inhibition of ARG by INCB001158 prevents tumor cells from utilizing arginine for polyamine generation. In combination with immune checkpoint therapy, INCB001158 has been used in patients with advanced/metastatic solid tumors and has shown significant efficacy in a Phase II clinical trial. Additionally, previous study has proved that ASS1 is a new tumor repressor via epigenetic mechanism and downregulated in cancers such as ovarian cancer. Its downregulation has been reported to be associated with advanced tumor stage and susceptible to ADI-PEG20 treatment [224].

Similarly, as mentioned above, tumors driven by *Myc* or *Kras* are highly dependent on exogenous glutamine, and pharmacological inhibitors of GLS like telaglenastat or 968 has shown excellent results in multiple tumors [7]. Besides, tumors control related enzymes expression to exploit serine and other one-carbon biosynthetic pathway to reduce the dependence on exogenous supply. Drugs targeting the one-carbon metabolic pathway like methotrexate targeting dihydrofolate reductase (DHFR) or 5-fluorouracil targeting thymidylate synthase has been widely used clinically.

### Amino acids modified dietary

Cumulating researches have demonstrated that amino acid like methionine, serine, glycine, leucine, glutamine and cysteine restriction plays roles in cancer intervention. Restricted methionine intake prevents tumor growth and metastasis such as TNBC [225], CRC [226], and glioma [227]. Preclinical studies show that restriction of serine and glycine intake significantly inhibits tumor cells proliferation in intestinal cancer and lymphoma mice [228]. In the leucine-rich diet, metabolism shifts from glycolytic metabolism to oxidative phosphorylation, resulting in a less aggressive tumor phenotype [229]. Metabolomic analysis reveals that dietary uptake of glutamine effectively increases the concentration of glutamine in tumors and its downstream metabolite, α-KG, without increasing biosynthetic intermediates necessary for cell proliferation. The increase in intratumoural α-KG concentration drives hypomethylation of H3K4me3, thereby suppressing epigenetically-activated oncogenic pathways in melanoma [230]. However, this effect may be melanoma specific, as glutamine would fuel tumor cell proliferation in most tumors.

Amino acid dietary restriction also affects tumor development by affecting immune cell function. Diet lack of sulfur-containing amino acids like methionine and cysteine downregulates the differentiation of CD4^+^ T cells towards Tregs and promotes the migration of CD8^+^ T cells, increasing the ratio of CD8^+^/Treg in tumors and thereby enhancing the immune killing function [231]. Dietary methionine restriction blocks cGAS methylation, releasing cGAS from chromatin and promoting it into cytoplasm [232]. Cytoplasm cGAS would sense dsDNA and activate the immune response, leading to tumor restriction. The effect of tumor immunotherapy is significantly enhanced by restricting the dose of amino acids in diet.

## Summary

During the process of proliferation, tumor cells dysregulate their metabolism to obtain more energy and nutrients. Amino acids are used as fuels and raw materials in protein synthesis as well as signal molecules in energy metabolism, participating in tumor growth process. In addition, amino acid metabolism also intertwines with signaling pathways, TME and epigenetic modification. Amino acids act as pivotal signaling molecules, stimulating signal pathways like mTORC1, MYC and KRAS to drive tumor growth and proliferation. Within the TME, a dynamic interplay occurs as nutrient competition and amino acid availability dictate tumor progression. Tumor and immune cells collaborate to regulate amino acid metabolism, which is crucial for sustaining the metabolic needs of proliferating tumor cells and sculpting the immune response. Moreover, amino acid-derived metabolites like α-KG, acetyl-CoA, NAD^+^ and SAM wield influence over epigenetic regulation. Comprehending this metabolism network provides foundation for targeted therapeutic approaches which aim to disrupt amino acid metabolism.

Compared with normal cells, tumor cells have more emphasis on amino acid requirement and dependence. Therefore, targeting amino acid metabolism to treat tumors is more effective and less damage. However, the field still faces many challenges. Some drugs targeting amino acid metabolism have shown promising effects in animal experiments but realize them clinically is still difficult. Insufficient amino acid depletion may merely maintain tumor cells in a state of cell quiescence, and once treatment is terminated, tumor cells will reappear. The effect of amino acid intervention on tumor development is not only related to tumor type but also depends on external factors even tumor location [233]. Since most metabolism inhibitors are not effective as single agents, combination therapy may be a more reasonable strategy. Nevertheless, compared with other therapies, amino acid depletion therapy is safer to normal cells. It is hoped that with the wider understanding of amino acid metabolism in tumor and the advancement of metabolic analysis techniques, appropriate patients can be identified. Thus, fully understand of the metabolic flexibility in amino acid metabolism in cancer cells is of great significance, which provides further insight into metabolic dependences and liabilities that can be exploited therapeutically.

### Supplementary information

checklist

## References

[1] Pavlova NN, Thompson CB (2016). The emerging hallmarks of cancer metabolism. Cell Metab.

[2] Sivanand S, Vander Heiden MG (2020). Emerging roles for branched-chain amino acid metabolism in cancer. Cancer Cell.

[3] Zhang Y, Morar M, Ealick SE (2008). Structural biology of the purine biosynthetic pathway. Cell Mol Life Sci.

[4] Morita M, Kudo K, Shima H, Tanuma N (2021). Dietary intervention as a therapeutic for cancer. Cancer Sci.

[5] Wei Z, Liu X, Cheng C, Yu W, Yi P (2020). Metabolism of amino acids in cancer. Front Cell Dev Biol.

[6] Taniguchi S, Elhance A, Van Duzer A, Kumar S, Leitenberger JJ, Oshimori N (2020). Tumor-initiating cells establish an IL-33-TGF-β niche signaling loop to promote cancer progression. Science.

[7] Butler M, van der Meer LT, van Leeuwen FN (2021). Amino acid depletion therapies: starving cancer cells to death. Trends Endocrinol Metab.

[8] Ji Y, Wu Z, Dai Z, Sun K, Wang J, Wu G (2016). Nutritional epigenetics with a focus on amino acids: implications for the development and treatment of metabolic syndrome. J Nutr Biochem.

[9] Vettore L, Westbrook RL, Tennant DA (2020). New aspects of amino acid metabolism in cancer. Br J Cancer.

[10] Lieu EL, Nguyen T, Rhyne S, Kim J (2020). Amino acids in cancer. Exp Mol Med.

[11] Lukey MJ, Katt WP, Cerione RA (2017). Targeting amino acid metabolism for cancer therapy. Drug Discov Today.

[12] Wise DR, DeBerardinis RJ, Mancuso A, Sayed N, Zhang X-Y, Pfeiffer HK (2008). Myc regulates a transcriptional program that stimulates mitochondrial glutaminolysis and leads to glutamine addiction. Proc Natl Acad Sci USA.

[13] Patel D, Menon D, Bernfeld E, Mroz V, Kalan S, Loayza D (2016). Aspartate rescues s-phase arrest caused by suppression of glutamine utilization in KRas-driven cancer cells. J Biol Chem.

[14] Luengo A, Gui DY, Vander Heiden MG (2017). Targeting metabolism for cancer therapy. Cell Chem Biol.

[15] Reynolds MR, Lane AN, Robertson B, Kemp S, Liu Y, Hill BG (2014). Control of glutamine metabolism by the tumor suppressor Rb. Oncogene.

[16] Wang Q, Tiffen J, Bailey CG, Lehman ML, Ritchie W, Fazli L (2013). Targeting amino acid transport in metastatic castration-resistant prostate cancer: effects on cell cycle, cell growth, and tumor development. J Natl Cancer Inst.

[17] Ren P, Yue M, Xiao D, Xiu R, Gan L, Liu H (2015). ATF4 and N-Myc coordinate glutamine metabolism in MYCN-amplified neuroblastoma cells through ASCT2 activation. J Pathol.

[18] Willems L, Jacque N, Jacquel A, Neveux N, Maciel TT, Lambert M (2013). Inhibiting glutamine uptake represents an attractive new strategy for treating acute myeloid leukemia. Blood.

[19] Lu J, Chen M, Tao Z, Gao S, Li Y, Cao Y (2017). Effects of targeting SLC1A5 on inhibiting gastric cancer growth and tumor development in vitro and in vivo. Oncotarget.

[20] Wang Q, Hardie RA, Hoy AJ, van Geldermalsen M, Gao D, Fazli L (2015). Targeting ASCT2-mediated glutamine uptake blocks prostate cancer growth and tumour development. J Pathol.

[21] van Geldermalsen M, Wang Q, Nagarajah R, Marshall AD, Thoeng A, Gao D (2016). ASCT2/SLC1A5 controls glutamine uptake and tumour growth in triple-negative basal-like breast cancer. Oncogene.

[22] Zhao J-S, Shi S, Qu H-Y, Keckesova Z, Cao Z-J, Yang L-X (2022). Glutamine synthetase licenses APC/C-mediated mitotic progression to drive cell growth. Nat Metab.

[23] Wang Z, Liu F, Fan N, Zhou C, Li D, Macvicar T (2020). Targeting glutaminolysis: new perspectives to understand cancer development and novel strategies for potential target therapies. Front Oncol.

[24] Ramirez-Peña E, Arnold J, Shivakumar V, Joseph R, Vidhya Vijay G, den Hollander P (2019). The epithelial to mesenchymal transition promotes glutamine independence by suppressing expression. Cancers.

[25] Suzuki S, Venkatesh D, Kanda H, Nakayama A, Hosokawa H, Lee E (2022). GLS2 is a tumor suppressor and a regulator of ferroptosis in hepatocellular carcinoma. Cancer Res.

[26] Xiang L, Mou J, Shao B, Wei Y, Liang H, Takano N (2019). Glutaminase 1 expression in colorectal cancer cells is induced by hypoxia and required for tumor growth, invasion, and metastatic colonization. Cell Death Dis.

[27] Gao P, Tchernyshyov I, Chang TC, Lee YS, Kita K, Ochi T (2009). c-Myc suppression of miR-23a/b enhances mitochondrial glutaminase expression and glutamine metabolism. Nature.

[28] Wang JB, Erickson JW, Fuji R, Ramachandran S, Gao P, Dinavahi R (2010). Targeting mitochondrial glutaminase activity inhibits oncogenic transformation. Cancer Cell.

[29] Kahlert UD, Cheng M, Koch K, Marchionni L, Fan X, Raabe EH (2016). Alterations in cellular metabolome after pharmacological inhibition of Notch in glioblastoma cells. Int J Cancer.

[30] Matés JM, Pérez-Gómez C, Núñez de Castro I, Asenjo M, Márquez J (2002). Glutamine and its relationship with intracellular redox status, oxidative stress and cell proliferation/death. Int J Biochem Cell Biol.

[31] Bott AJ, Maimouni S, Zong W-X (2019). The Pleiotropic effects of glutamine metabolism in cancer. Cancers.

[32] Fu S, Li Z, Xiao L, Hu W, Zhang L, Xie B (2019). Glutamine synthetase promotes radiation resistance via facilitating nucleotide metabolism and subsequent DNA damage repair. Cell Rep..

[33] Vincent EE, Sergushichev A, Griss T, Gingras M-C, Samborska B, Ntimbane T (2015). Mitochondrial Phosphoenolpyruvate carboxykinase regulates metabolic adaptation and enables glucose-independent tumor growth. Mol Cell.

[34] Lio C-WJ, Yuita H, Rao A (2019). Dysregulation of the TET family of epigenetic regulators in lymphoid and myeloid malignancies. Blood.

[35] Ji JX, Cochrane DR, Tessier-Cloutier B, Chen SY, Ho G, Pathak KV (2020). Arginine depletion therapy with ADI-PEG20 limits tumor growth in argininosuccinate synthase-deficient ovarian cancer, including small-cell carcinoma of the ovary, hypercalcemic type. Clin Cancer Res.

[36] Lee JS, Adler L, Karathia H, Carmel N, Rabinovich S, Auslander N (2018). Urea cycle dysregulation generates clinically relevant genomic and biochemical signatures. Cell.

[37] Scalise M, Console L, Rovella F, Galluccio M, Pochini L, Indiveri C (2020). Membrane transporters for amino acids as players of cancer metabolic rewiring. Cells.

[38] Abdelmagid SA, Rickard JA, McDonald WJ, Thomas LN, Too CKL (2011). CAT-1-mediated arginine uptake and regulation of nitric oxide synthases for the survival of human breast cancer cell lines. J Cell Biochem.

[39] Bachmann AS, Geerts D (2018). Polyamine synthesis as a target of MYC oncogenes. J Biol Chem.

[40] Grzywa TM, Sosnowska A, Matryba P, Rydzynska Z, Jasinski M, Nowis D (2020). Myeloid cell-derived arginase in cancer immune response. Front Immunol.

[41] Kryczek I, Zou L, Rodriguez P, Zhu G, Wei S, Mottram P (2006). B7-H4 expression identifies a novel suppressive macrophage population in human ovarian carcinoma. J Exp Med.

[42] Novita Sari I, Setiawan T, Seock Kim K, Toni Wijaya Y, Won Cho K, Young Kwon H (2021). Metabolism and function of polyamines in cancer progression. Cancer Lett.

[43] Ha HC, Sirisoma NS, Kuppusamy P, Zweier JL, Woster PM, Casero RA (1998). The natural polyamine spermine functions directly as a free radical scavenger. Proc Natl Acad Sci USA.

[44] Ha HC, Yager JD, Woster PA, Casero RA (1998). Structural specificity of polyamines and polyamine analogues in the protection of DNA from strand breaks induced by reactive oxygen species. Biochem Biophys Res Commun.

[45] Palmer RM, Ashton DS, Moncada S (1988). Vascular endothelial cells synthesize nitric oxide from L-arginine. Nature.

[46] Brito C, Naviliat M, Tiscornia AC, Vuillier F, Gualco G, Dighiero G (1999). Peroxynitrite inhibits T lymphocyte activation and proliferation by promoting impairment of tyrosine phosphorylation and peroxynitrite-driven apoptotic death. J Immunol.

[47] Singh N, Ecker GF (2018). Insights into the structure, function, and ligand discovery of the large neutral amino acid Transporter 1, LAT1. Int J Mol Sci.

[48] Wang Q, Holst J (2015). L-type amino acid transport and cancer: targeting the mTORC1 pathway to inhibit neoplasia. Am J Cancer Res.

[49] Zhang B, Chen Y, Shi X, Zhou M, Bao L, Hatanpaa KJ (2021). Regulation of branched-chain amino acid metabolism by hypoxia-inducible factor in glioblastoma. Cell Mol Life Sci.

[50] Elorza A, Soro-Arnáiz I, Meléndez-Rodríguez F, Rodríguez-Vaello V, Marsboom G, de Cárcer G (2012). HIF2α acts as an mTORC1 activator through the amino acid carrier SLC7A5. Mol Cell.

[51] Quanz M, Bender E, Kopitz C, Grünewald S, Schlicker A, Schwede W (2018). Preclinical efficacy of the novel monocarboxylate Transporter 1 inhibitor BAY-8002 and associated markers of resistance. Mol Cancer Ther.

[52] Zhao X, Sakamoto S, Wei J, Pae S, Saito S, Sazuka T (2023). Contribution of the L-Type amino acid transporter family in the diagnosis and treatment of prostate cancer. Int J Mol Sci.

[53] Ananieva EA, Wilkinson AC (2018). Branched-chain amino acid metabolism in cancer. Curr Opin Clin Nutr Metab Care.

[54] Ericksen RE, Lim SL, McDonnell E, Shuen WH, Vadiveloo M, White PJ (2019). Loss of BCAA catabolism during carcinogenesis enhances mTORC1 activity and promotes tumor development and progression. Cell Metab.

[55] Tian T, Li X, Zhang J (2019). mTOR signaling in cancer and mTOR inhibitors in solid tumor targeting therapy. Int J Mol Sci.

[56] Kamphorst JJ, Nofal M, Commisso C, Hackett SR, Lu W, Grabocka E (2015). Human pancreatic cancer tumors are nutrient poor and tumor cells actively scavenge extracellular protein. Cancer Res.

[57] Cano-Crespo S, Chillarón J, Junza A, Fernández-Miranda G, García J, Polte C (2019). CD98hc (SLC3A2) sustains amino acid and nucleotide availability for cell cycle progression. Sci Rep..

[58] Nordlund P, Reichard P (2006). Ribonucleotide reductases. Annu Rev Biochem.

[59] Mayers JR, Wu C, Clish CB, Kraft P, Torrence ME, Fiske BP (2014). Elevation of circulating branched-chain amino acids is an early event in human pancreatic adenocarcinoma development. Nat Med.

[60] Liu X-H, Zhai X-Y (2021). Role of tryptophan metabolism in cancers and therapeutic implications. Biochimie.

[61] Della Chiesa M, Carlomagno S, Frumento G, Balsamo M, Cantoni C (2006). The tryptophan catabolite L-kynurenine inhibits the surface expression of NKp46- and NKG2D-activating receptors and regulates NK-cell function. Blood.

[62] Abd El-Fattah EE (2022). IDO/kynurenine pathway in cancer: possible therapeutic approaches. J Transl Med.

[63] Xue C, Li G, Zheng Q, Gu X, Shi Q, Su Y (2023). Tryptophan metabolism in health and disease. Cell Metab.

[64] Tummala KS, Gomes AL, Yilmaz M, Graña O, Bakiri L, Ruppen I (2014). Inhibition of de novo NAD(+) synthesis by oncogenic URI causes liver tumorigenesis through DNA damage. Cancer Cell.

[65] Muthusamy T, Cordes T, Handzlik MK, You L, Lim EW, Gengatharan J (2020). Serine restriction alters sphingolipid diversity to constrain tumour growth. Nature.

[66] Platten M, Nollen EAA, Röhrig UF, Fallarino F, Opitz CA (2019). Tryptophan metabolism as a common therapeutic target in cancer, neurodegeneration and beyond. Nat Rev Drug Discov.

[67] Sarrouilhe D, Mesnil M (2019). Serotonin and human cancer: a critical view. Biochimie.

[68] Schneider MA, Heeb L, Beffinger MM, Pantelyushin S, Linecker M, Roth L (2021). Attenuation of peripheral serotonin inhibits tumor growth and enhances immune checkpoint blockade therapy in murine tumor models. Sci Transl Med.

[69] Hezaveh K, Shinde RS, Klötgen A, Halaby MJ, Lamorte S, Ciudad MT (2022). Tryptophan-derived microbial metabolites activate the aryl hydrocarbon receptor in tumor-associated macrophages to suppress anti-tumor immunity. Immunity.

[70] Jia Y, Wang H, Wang Y, Wang T, Wang M, Ma M (2015). Low expression of Bin1, along with high expression of IDO in tumor tissue and draining lymph nodes, are predictors of poor prognosis for esophageal squamous cell cancer patients. Int J Cancer.

[71] Mellor AL, Lemos H, Huang L (2017). Indoleamine 2,3-Dioxygenase and tolerance: where are we now?. Front Immunol.

[72] Kindler J, Lim CK, Weickert CS, Boerrigter D, Galletly C, Liu D (2020). Dysregulation of kynurenine metabolism is related to proinflammatory cytokines, attention, and prefrontal cortex volume in schizophrenia. Mol Psychiatry.

[73] Brandacher G, Perathoner A, Ladurner R, Schneeberger S, Obrist P, Winkler C (2006). Prognostic value of indoleamine 2,3-dioxygenase expression in colorectal cancer: effect on tumor-infiltrating T cells. Clin Cancer Res.

[74] Tang D, Yue L, Yao R, Zhou L, Yang Y, Lu L (2017). P53 prevent tumor invasion and metastasis by down-regulating IDO in lung cancer. Oncotarget.

[75] Hsu YL, Hung JY, Chiang SY, Jian SF, Wu CY, Lin YS (2016). Lung cancer-derived galectin-1 contributes to cancer associated fibroblast-mediated cancer progression and immune suppression through TDO2/kynurenine axis. Oncotarget.

[76] Garcia-Bermudez J, Baudrier L, La K, Zhu XG, Fidelin J, Sviderskiy VO (2018). Aspartate is a limiting metabolite for cancer cell proliferation under hypoxia and in tumours. Nat Cell Biol.

[77] Birsoy K, Wang T, Chen WW, Freinkman E, Abu-Remaileh M, Sabatini DM (2015). An essential role of the mitochondrial electron transport chain in cell proliferation is to enable aspartate synthesis. Cell.

[78] Sullivan LB, Luengo A, Danai LV, Bush LN, Diehl FF, Hosios AM (2018). Aspartate is an endogenous metabolic limitation for tumour growth. Nat Cell Biol.

[79] Krall AS, Xu S, Graeber TG, Braas D, Christofk HR (2016). Asparagine promotes cancer cell proliferation through use as an amino acid exchange factor. Nat Commun.

[80] Knott SRV, Wagenblast E, Khan S, Kim SY, Soto M, Wagner M (2018). Asparagine bioavailability governs metastasis in a model of breast cancer. Nature.

[81] Luo M, Brooks M, Wicha MS (2018). Asparagine and glutamine: co-conspirators fueling metastasis. Cell Metab.

[82] Pavlova NN, Hui S, Ghergurovich JM, Fan J, Intlekofer AM, White RM (2018). As extracellular glutamine levels decline, asparagine becomes an essential amino acid. Cell Metab.

[83] Lee GY, Haverty PM, Li L, Kljavin NM, Bourgon R, Lee J (2014). Comparative oncogenomics identifies PSMB4 and SHMT2 as potential cancer driver genes. Cancer Res.

[84] Jain M, Nilsson R, Sharma S, Madhusudhan N, Kitami T, Souza AL (2012). Metabolite profiling identifies a key role for glycine in rapid cancer cell proliferation. Science.

[85] Nilsson R, Jain M, Madhusudhan N, Sheppard NG, Strittmatter L, Kampf C (2014). Metabolic enzyme expression highlights a key role for MTHFD2 and the mitochondrial folate pathway in cancer. Nat Commun.

[86] Maddocks ODK, Labuschagne CF, Adams PD, Vousden KH (2016). Serine metabolism supports the methionine cycle and DNA/RNA Methylation through De Novo ATP synthesis in cancer cells. Mol Cell.

[87] Wei Z, Song J, Wang G, Cui X, Zheng J, Tang Y (2018). Deacetylation of serine hydroxymethyl-transferase 2 by SIRT3 promotes colorectal carcinogenesis. Nat Commun.

[88] Wan X, Wang C, Huang Z, Zhou D, Xiang S, Qi Q (2020). Cisplatin inhibits SIRT3-deacetylation MTHFD2 to disturb cellular redox balance in colorectal cancer cell. Cell Death Dis.

[89] Wang C, Wan X, Yu T, Huang Z, Shen C, Qi Q (2020). Acetylation stabilizes phosphoglycerate dehydrogenase by disrupting the interaction of E3 ligase RNF5 to promote breast tumorigenesis. Cell Rep..

[90] Tajan M, Hennequart M, Cheung EC, Zani F, Hock AK, Legrave N (2021). Serine synthesis pathway inhibition cooperates with dietary serine and glycine limitation for cancer therapy. Nat Commun.

[91] Parsa S, Ortega-Molina A, Ying HY, Jiang M, Teater M, Wang J (2020). The serine hydroxymethyltransferase-2 (SHMT2) initiates lymphoma development through epigenetic tumor suppressor silencing. Nat Cancer.

[92] Xia J, Zhang J, Wu X, Du W, Zhu Y, Liu X (2022). Blocking glycine utilization inhibits multiple myeloma progression by disrupting glutathione balance. Nat Commun.

[93] Liu C, Zou W, Nie D, Li S, Duan C, Zhou M (2022). Loss of PRMT7 reprograms glycine metabolism to selectively eradicate leukemia stem cells in CML. Cell Metab.

[94] Saxton RA, Sabatini DM (2017). mTOR signaling in growth, metabolism, and disease. Cell.

[95] Karlsson E, Pérez-Tenorio G, Amin R, Bostner J, Skoog L, Fornander T (2013). The mTOR effectors 4EBP1 and S6K2 are frequently coexpressed, and associated with a poor prognosis and endocrine resistance in breast cancer: a retrospective study including patients from the randomised Stockholm tamoxifen trials. Breast Cancer Res.

[96] Laplante M, Sabatini DM (2012). mTOR signaling in growth control and disease. Cell.

[97] Jewell JL, Russell RC, Guan K-L (2013). Amino acid signalling upstream of mTOR. Nat Rev Mol Cell Biol.

[98] He X-D, Gong W, Zhang J-N, Nie J, Yao C-F, Guo F-S (2018). Sensing and transmitting intracellular amino acid signals through reversible lysine aminoacylations. Cell Metab.

[99] Chen J, Ou Y, Luo R, Wang J, Wang D, Guan J (2021). SAR1B senses leucine levels to regulate mTORC1 signalling. Nature.

[100] Durán RV, Oppliger W, Robitaille AM, Heiserich L, Skendaj R, Gottlieb E (2012). Glutaminolysis activates Rag-mTORC1 signaling. Mol Cell.

[101] Gu X, Orozco JM, Saxton RA, Condon KJ, Liu GY, Krawczyk PA (2017). SAMTOR is an -adenosylmethionine sensor for the mTORC1 pathway. Science.

[102] Chen J, Ou Y, Yang Y, Li W, Xu Y, Xie Y (2018). KLHL22 activates amino-acid-dependent mTORC1 signalling to promote tumorigenesis and ageing. Nature.

[103] Han JM, Jeong SJ, Park MC, Kim G, Kwon NH, Kim HK (2012). Leucyl-tRNA synthetase is an intracellular leucine sensor for the mTORC1-signaling pathway. Cell.

[104] Mossmann D, Park S, Hall MN (2018). mTOR signalling and cellular metabolism are mutual determinants in cancer. Nat Rev Cancer.

[105] Bar-Peled L, Schweitzer LD, Zoncu R, Sabatini DM (2012). Ragulator is a GEF for the rag GTPases that signal amino acid levels to mTORC1. Cell.

[106] Wyant GA, Abu-Remaileh M, Wolfson RL, Chen WW, Freinkman E, Danai LV (2017). mTORC1 activator SLC38A9 is required to efflux essential amino acids from lysosomes and use protein as a nutrient. Cell.

[107] Shen K, Sabatini DM (2018). Ragulator and SLC38A9 activate the Rag GTPases through noncanonical GEF mechanisms. Proc Natl Acad Sci USA.

[108] Wang S, Tsun ZY, Wolfson RL, Shen K, Wyant GA, Plovanich ME (2015). Metabolism. Lysosomal amino acid transporter SLC38A9 signals arginine sufficiency to mTORC1. Science.

[109] Ben-Sahra I, Hoxhaj G, Ricoult SJH, Asara JM, Manning BD (2016). mTORC1 induces purine synthesis through control of the mitochondrial tetrahydrofolate cycle. Science.

[110] Zhu J, Thompson CB (2019). Metabolic regulation of cell growth and proliferation. Nat Rev Mol Cell Biol.

[111] Simcox J, Lamming DW (2022). The central moTOR of metabolism. Dev Cell.

[112] Csibi A, Fendt S-M, Li C, Poulogiannis G, Choo AY, Chapski DJ (2013). The mTORC1 pathway stimulates glutamine metabolism and cell proliferation by repressing SIRT4. Cell.

[113] Origanti S, Nowotarski SL, Carr TD, Sass-Kuhn S, Xiao L, Wang JY (2012). Ornithine decarboxylase mRNA is stabilized in an mTORC1-dependent manner in Ras-transformed cells. Biochem J.

[114] Tsai WB, Aiba I, Long Y, Lin HK, Feun L, Savaraj N (2012). Activation of Ras/PI3K/ERK pathway induces c-Myc stabilization to upregulate argininosuccinate synthetase, leading to arginine deiminase resistance in melanoma cells. Cancer Res.

[115] Wahlström T, Henriksson MA (2015). Impact of MYC in regulation of tumor cell metabolism. Biochim Biophys Acta.

[116] Huang H, Weng H, Zhou H, Qu L (2014). Attacking c-Myc: targeted and combined therapies for cancer. Curr Pharm Des.

[117] Babu E, Kanai Y, Chairoungdua A, Kim DK, Iribe Y, Tangtrongsup S (2003). Identification of a novel system L amino acid transporter structurally distinct from heterodimeric amino acid transporters. J Biol Chem.

[118] Kanai Y, Segawa H, Miyamoto K, Uchino H, Takeda E, Endou H (1998). Expression cloning and characterization of a transporter for large neutral amino acids activated by the heavy chain of 4F2 antigen (CD98). J Biol Chem.

[119] Yue M, Jiang J, Gao P, Liu H, Qing G (2017). Oncogenic MYC activates a feedforward regulatory loop promoting essential amino acid metabolism and tumorigenesis. Cell Rep..

[120] Venkateswaran N, Lafita-Navarro MC, Hao YH, Kilgore JA, Perez-Castro L, Braverman J (2019). MYC promotes tryptophan uptake and metabolism by the kynurenine pathway in colon cancer. Genes Dev.

[121] Dong Y, Tu R, Liu H, Qing G (2020). Regulation of cancer cell metabolism: oncogenic MYC in the driver’s seat. Signal Transduct Target Ther.

[122] Cervenka I, Agudelo LZ, Ruas JL (2017). Kynurenines: tryptophan’s metabolites in exercise, inflammation, and mental health. Science.

[123] El Ansari R, McIntyre A, Craze ML, Ellis IO, Rakha EA, Green AR (2018). Altered glutamine metabolism in breast cancer; subtype dependencies and alternative adaptations. Histopathology.

[124] Bott AJ, Peng IC, Fan Y, Faubert B, Zhao L, Li J (2015). Oncogenic Myc induces expression of glutamine synthetase through promoter demethylation. Cell Metab.

[125] Geng P, Qin W, Xu G (2021). Proline metabolism in cancer. Amino Acids.

[126] Liu W, Zabirnyk O, Wang H, Shiao YH, Nickerson ML, Khalil S (2010). miR-23b targets proline oxidase, a novel tumor suppressor protein in renal cancer. Oncogene.

[127] Liu W, Le A, Hancock C, Lane AN, Dang CV, Fan TWM (2012). Reprogramming of proline and glutamine metabolism contributes to the proliferative and metabolic responses regulated by oncogenic transcription factor c-MYC. Proc Natl Acad Sci USA.

[128] Xie M, Pei D-S (2021). Serine hydroxymethyltransferase 2: a novel target for human cancer therapy. Investig N. Drugs.

[129] Prior IA, Hood FE, Hartley JL (2020). The frequency of ras mutations in cancer. Cancer Res.

[130] Fruman DA, Chiu H, Hopkins BD, Bagrodia S, Cantley LC, Abraham RT (2017). The PI3K pathway in human disease. Cell.

[131] Spiegel J, Cromm PM, Zimmermann G, Grossmann TN, Waldmann H (2014). Small-molecule modulation of Ras signaling. Nat Chem Biol.

[132] Chakrabarti G, Gerber DE, Boothman DA (2015). Expanding antitumor therapeutic windows by targeting cancer-specific nicotinamide adenine dinucleotide phosphate-biogenesis pathways. Clin Pharm.

[133] Guo JY, Chen H-Y, Mathew R, Fan J, Strohecker AM, Karsli-Uzunbas G (2011). Activated Ras requires autophagy to maintain oxidative metabolism and tumorigenesis. Genes Dev.

[134] Hinshaw DC, Shevde LA (2019). The tumor microenvironment innately modulates cancer progression. Cancer Res.

[135] Swanson JA, Watts C (1995). Macropinocytosis. Trends Cell Biol.

[136] Palm W, Araki J, King B, DeMatteo RG, Thompson CB (2017). Critical role for PI3-kinase in regulating the use of proteins as an amino acid source. Proc Natl Acad Sci USA.

[137] Recouvreux MV, Commisso C (2017). Macropinocytosis: a metabolic adaptation to nutrient stress in cancer. Front Endocrinol.

[138] Zhang YF, Li Q, Huang PQ, Su T, Jiang SH, Hu LP (2022). A low amino acid environment promotes cell macropinocytosis through the YY1-FGD6 axis in Ras-mutant pancreatic ductal adenocarcinoma. Oncogene.

[139] Corbet C, Draoui N, Polet F, Pinto A, Drozak X, Riant O (2014). The SIRT1/HIF2α axis drives reductive glutamine metabolism under chronic acidosis and alters tumor response to therapy. Cancer Res.

[140] Powell DW, Mifflin RC, Valentich JD, Crowe SE, Saada JI, West AB (1999). Myofibroblasts. I. Paracrine cells important in health and disease. Am J Physiol.

[141] Chen X, Song E (2019). Turning foes to friends: targeting cancer-associated fibroblasts. Nat Rev Drug Discov.

[142] Tajan M, Hock AK, Blagih J, Robertson NA, Labuschagne CF, Kruiswijk F (2018). A role for p53 in the adaptation to glutamine starvation through the expression of SLC1A3. Cell Metab.

[143] Bertero T, Oldham WM, Grasset EM, Bourget I, Boulter E, Pisano S (2019). Tumor-stroma mechanics coordinate amino acid availability to sustain tumor growth and malignancy. Cell Metab.

[144] Liu T, Han C, Fang P, Ma Z, Wang X, Chen H (2022). Cancer-associated fibroblast-specific lncRNA LINC01614 enhances glutamine uptake in lung adenocarcinoma. J Hematol Oncol.

[145] Schwörer S, Berisa M, Violante S, Qin W, Zhu J, Hendrickson RC (2020). Proline biosynthesis is a vent for TGFβ-induced mitochondrial redox stress. Embo j.

[146] Geiger R, Rieckmann JC, Wolf T, Basso C, Feng Y, Fuhrer T (2016). L-Arginine modulates T cell metabolism and enhances survival and anti-tumor activity. Cell.

[147] Consonni FM, Porta C, Marino A, Pandolfo C, Mola S, Bleve A (2019). Myeloid-derived suppressor cells: ductile targets in disease. Front Immunol.

[148] Srivastava MK, Sinha P, Clements VK, Rodriguez P, Ostrand-Rosenberg S (2010). Myeloid-derived suppressor cells inhibit T-cell activation by depleting cystine and cysteine. Cancer Res.

[149] Baumjohann D, Kageyama R, Clingan JM, Morar MM, Patel S, de Kouchkovsky D (2013). The microRNA cluster miR-17∼92 promotes TFH cell differentiation and represses subset-inappropriate gene expression. Nat Immunol.

[150] Mantovani A, Marchesi F, Malesci A, Laghi L, Allavena P (2017). Tumour-associated macrophages as treatment targets in oncology. Nat Rev Clin Oncol.

[151] Palmieri EM, Menga A, Martín-Pérez R, Quinto A, Riera-Domingo C, De Tullio G (2017). Pharmacologic or genetic targeting of glutamine synthetase skews macrophages toward an M1-like phenotype and inhibits tumor metastasis. Cell Rep..

[152] Ma G, Zhang Z, Li P, Zhang Z, Zeng M, Liang Z (2022). Reprogramming of glutamine metabolism and its impact on immune response in the tumor microenvironment. Cell Commun Signal.

[153] Noy R, Pollard JW (2014). Tumor-associated macrophages: from mechanisms to therapy. Immunity.

[154] Wang W, Zou W (2020). Amino acids and their transporters in T cell immunity and cancer therapy. Mol Cell.

[155] Verbist KC, Guy CS, Milasta S, Liedmann S, Kamiński MM, Wang R (2016). Metabolic maintenance of cell asymmetry following division in activated T lymphocytes. Nature.

[156] Stepka P, Vsiansky V, Raudenska M, Gumulec J, Adam V, Masarik M (2021). Metabolic and amino acid alterations of the tumor microenvironment. Curr Med Chem.

[157] Klysz D, Tai X, Robert PA, Craveiro M, Cretenet G, Oburoglu L (2015). Glutamine-dependent α-ketoglutarate production regulates the balance between T helper 1 cell and regulatory T cell generation. Sci Signal.

[158] Munn DH, Sharma MD, Baban B, Harding HP, Zhang Y, Ron D (2005). GCN2 kinase in T cells mediates proliferative arrest and anergy induction in response to indoleamine 2,3-dioxygenase. Immunity.

[159] Munn DH, Mellor AL (2013). Indoleamine 2,3 dioxygenase and metabolic control of immune responses. Trends Immunol.

[160] Mezrich JD, Fechner JH, Zhang X, Johnson BP, Burlingham WJ, Bradfield CA (2010). An interaction between kynurenine and the aryl hydrocarbon receptor can generate regulatory T cells. J Immunol.

[161] Guo C, You Z, Shi H, Sun Y, Du X, Palacios G (2023). SLC38A2 and glutamine signalling in cDC1s dictate anti-tumour immunity. Nature.

[162] Corsale AM, Di Simone M, Lo Presti E, Picone C, Dieli F, Meraviglia S (2021). Metabolic changes in tumor microenvironment: how could they affect γδ T cells functions?. Cells.

[163] Douguet L, Bod L, Lengagne R, Labarthe L, Kato M, Avril MF (2016). Nitric oxide synthase 2 is involved in the pro-tumorigenic potential of γδ17 T cells in melanoma. Oncoimmunology.

[164] Wu J, Li G, Li L, Li D, Dong Z, Jiang P (2021). Asparagine enhances LCK signalling to potentiate CD8 T-cell activation and anti-tumour responses. Nat Cell Biol.

[165] Cavalli G, Heard E (2019). Advances in epigenetics link genetics to the environment and disease. Nature.

[166] Su X, Wellen KE, Rabinowitz JD (2016). Metabolic control of methylation and acetylation. Curr Opin Chem Biol.

[167] Mentch SJ, Locasale JW (2016). One-carbon metabolism and epigenetics: understanding the specificity. Ann N. Y Acad Sci.

[168] Michalak EM, Burr ML, Bannister AJ, Dawson MA (2019). The roles of DNA, RNA and histone methylation in ageing and cancer. Nat Rev Mol Cell Biol.

[169] Greer EL, Shi Y (2012). Histone methylation: a dynamic mark in health, disease and inheritance. Nat Rev Genet.

[170] Bian Y, Li W, Kremer DM, Sajjakulnukit P, Li S, Crespo J (2020). Cancer SLC43A2 alters T cell methionine metabolism and histone methylation. Nature.

[171] Cheng H, Qiu Y, Xu Y, Chen L, Ma K, Tao M (2023). Extracellular acidosis restricts one-carbon metabolism and preserves T cell stemness. Nat Metab.

[172] Dai Z, Mentch SJ, Gao X, Nichenametla SN, Locasale JW (2018). Methionine metabolism influences genomic architecture and gene expression through H3K4me3 peak width. Nat Commun.

[173] Shyh-Chang N, Locasale JW, Lyssiotis CA, Zheng Y, Teo RY, Ratanasirintrawoot S (2013). Influence of threonine metabolism on S-adenosylmethionine and histone methylation. Science.

[174] Obata F, Kuranaga E, Tomioka K, Ming M, Takeishi A, Chen C-H (2014). Necrosis-driven systemic immune response alters SAM metabolism through the FOXO-GNMT axis. Cell Rep..

[175] Melnyk S, Pogribna M, Pogribny IP, Yi P, James SJ (2000). Measurement of plasma and intracellular S-adenosylmethionine and S-adenosylhomocysteine utilizing coulometric electrochemical detection: alterations with plasma homocysteine and pyridoxal 5’-phosphate concentrations. Clin Chem.

[176] Martínez-Chantar ML, Vázquez-Chantada M, Ariz U, Martínez N, Varela M, Luka Z (2008). Loss of the glycine N-methyltransferase gene leads to steatosis and hepatocellular carcinoma in mice. Hepatology.

[177] Liao Y-J, Liu S-P, Lee C-M, Yen C-H, Chuang P-C, Chen C-Y (2009). Characterization of a glycine N-methyltransferase gene knockout mouse model for hepatocellular carcinoma: Implications of the gender disparity in liver cancer susceptibility. Int J Cancer.

[178] An J, Rao A, Ko M (2017). TET family dioxygenases and DNA demethylation in stem cells and cancers. Exp Mol Med.

[179] Baksh SC, Finley LWS (2021). Metabolic coordination of cell fate by α-Ketoglutarate-Dependent Dioxygenases. Trends Cell Biol.

[180] Huang J, Yu J, Tu L, Huang N, Li H, Luo Y (2019). Isocitrate Dehydrogenase Mutations In Glioma: From Basic Discovery To Therapeutics Development. Front Oncol.

[181] Tommasini-Ghelfi S, Murnan K, Kouri FM, Mahajan AS, May JL, Stegh AH (2019). Cancer-associated mutation and beyond: the emerging biology of isocitrate dehydrogenases in human disease. Sci Adv.

[182] Tönjes M, Barbus S, Park YJ, Wang W, Schlotter M, Lindroth AM (2013). BCAT1 promotes cell proliferation through amino acid catabolism in gliomas carrying wild-type IDH1. Nat Med.

[183] Raffel S, Falcone M, Kneisel N, Hansson J, Wang W, Lutz C (2017). BCAT1 restricts αKG levels in AML stem cells leading to IDHmut-like DNA hypermethylation. Nature.

[184] Mahlknecht U, Hoelzer D (2000). Histone acetylation modifiers in the pathogenesis of malignant disease. Mol Med.

[185] Lee JV, Carrer A, Shah S, Snyder NW, Wei S, Venneti S (2014). Akt-dependent metabolic reprogramming regulates tumor cell histone acetylation. Cell Metab.

[186] Park SY, Kim JS (2020). A short guide to histone deacetylases including recent progress on class II enzymes. Exp Mol Med.

[187] Son SM, Park SJ, Lee H, Siddiqi F, Lee JE, Menzies FM (2019). Leucine signals to mTORC1 via its metabolite acetyl-coenzyme A. Cell Metab.

[188] White PJ, McGarrah RW, Grimsrud PA, Tso S-C, Yang W-H, Haldeman JM (2018). The BCKDH kinase and phosphatase integrate BCAA and lipid metabolism via regulation of ATP-Citrate Lyase. Cell Metab.

[189] Kouzarides T (1999). Histone acetylases and deacetylases in cell proliferation. Curr Opin Genet Dev.

[190] Kebede AF, Schneider R, Daujat S (2015). Novel types and sites of histone modifications emerge as players in the transcriptional regulation contest. Febs j.

[191] Sun L, Zhang H, Gao P (2022). Metabolic reprogramming and epigenetic modifications on the path to cancer. Protein Cell.

[192] Herzig S, Shaw RJ (2018). AMPK: guardian of metabolism and mitochondrial homeostasis. Nat Rev Mol Cell Biol.

[193] Adachi Y, De Sousa-Coelho AL, Harata I, Aoun C, Weimer S, Shi X (2018). l-Alanine activates hepatic AMP-activated protein kinase and modulates systemic glucose metabolism. Mol Metab.

[194] Deng L, Yao P, Li L, Ji F, Zhao S, Xu C (2020). p53-mediated control of aspartate-asparagine homeostasis dictates LKB1 activity and modulates cell survival. Nat Commun.

[195] Cohen I, Poręba E, Kamieniarz K, Schneider R (2011). Histone modifiers in cancer: friends or foes?. Genes Cancer.

[196] Tian Q, Yuan P, Quan C, Li M, Xiao J, Zhang L (2020). Phosphorylation of BCKDK of BCAA catabolism at Y246 by Src promotes metastasis of colorectal cancer. Oncogene.

[197] Han T, Zhan W, Gan M, Liu F, Yu B, Chin YE (2018). Phosphorylation of glutaminase by PKCε is essential for its enzymatic activity and critically contributes to tumorigenesis. Cell Res.

[198] Smestad J, Erber L, Chen Y, Maher LJ (2018). Chromatin succinylation correlates with active gene expression and is perturbed by defective TCA cycle metabolism. iScience.

[199] Piñeiro M, González PJ, Hernández F, Palacián E (1991). Interaction of RNA polymerase II with acetylated nucleosomal core particles. Biochem Biophys Res Commun.

[200] Eniafe J, Jiang S (2021). The functional roles of TCA cycle metabolites in cancer. Oncogene.

[201] Tong Y, Guo D, Lin SH, Liang J, Yang D, Ma C (2021). SUCLA2-coupled regulation of GLS succinylation and activity counteracts oxidative stress in tumor cells. Mol Cell.

[202] Zhang D, Tang Z, Huang H, Zhou G, Cui C, Weng Y (2019). Metabolic regulation of gene expression by histone lactylation. Nature.

[203] Su J, Zheng Z, Bian C, Chang S, Bao J, Yu H (2023). Functions and mechanisms of lactylation in carcinogenesis and immunosuppression. Front Immunol.

[204] Xiong J, He J, Zhu J, Pan J, Liao W, Ye H (2022). Lactylation-driven METTL3-mediated RNA m(6)A modification promotes immunosuppression of tumor-infiltrating myeloid cells. Mol Cell.

[205] Fung MKL, Chan GC-F (2017). Drug-induced amino acid deprivation as strategy for cancer therapy. J Hematol Oncol.

[206] Bhutia YD, Babu E, Ramachandran S, Ganapathy V (2015). Amino acid transporters in cancer and their relevance to “glutamine addiction”: novel targets for the design of a new class of anticancer drugs. Cancer Res.

[207] Schulte ML, Fu A, Zhao P, Li J, Geng L, Smith ST (2018). Pharmacological blockade of ASCT2-dependent glutamine transport leads to antitumor efficacy in preclinical models. Nat Med.

[208] Cha YJ, Kim E-S, Koo JS (2018). Amino acid transporters and glutamine metabolism in breast cancer. Int J Mol Sci.

[209] Peng JB, Zhuang L, Berger UV, Adam RM, Williams BJ, Brown EM (2001). CaT1 expression correlates with tumor grade in prostate cancer. Biochem Biophys Res Commun.

[210] Lowman XH, Hanse EA, Yang Y, Ishak Gabra MB, Tran TQ, Li H (2019). p53 promotes cancer cell adaptation to glutamine deprivation by upregulating Slc7a3 to increase arginine uptake. Cell Rep..

[211] Werner A, Pieh D, Echchannaoui H, Rupp J, Rajalingam K, Theobald M (2019). Cationic amino acid Transporter-1-Mediated Arginine uptake is essential for chronic lymphocytic leukemia cell proliferation and viability. Front Oncol.

[212] Banjarnahor S, König J, Maas R (2022). Screening of commonly prescribed drugs for effects on the CAT1-mediated transport of L-arginine and arginine derivatives. Amino Acids.

[213] Coburn LA, Singh K, Asim M, Barry DP, Allaman MM, Al-Greene NT (2019). Loss of solute carrier family 7 member 2 exacerbates inflammation-associated colon tumorigenesis. Oncogene.

[214] Wang C, Wu J, Wang Z, Yang Z, Li Z, Deng H (2018). Glutamine addiction activates polyglutamine-based nanocarriers delivering therapeutic siRNAs to orthotopic lung tumor mediated by glutamine transporter SLC1A5. Biomaterials.

[215] Rogala-Koziarska K, Samluk Ł, Nałęcz KA (2019). Amino acid transporter SLC6A14 depends on heat shock protein HSP90 in trafficking to the cell surface. Biochim Biophys Acta Mol Cell Res.

[216] Luo Q, Yang B, Tao W, Li J, Kou L, Lian H (2017). ATB(0,+) transporter-mediated targeting delivery to human lung cancer cells via aspartate-modified docetaxel-loading stealth liposomes. Biomater Sci.

[217] Avramis VI, Tiwari PN (2006). Asparaginase (native ASNase or pegylated ASNase) in the treatment of acute lymphoblastic leukemia. Int J Nanomed.

[218] Van Trimpont M, Peeters E, De Visser Y, Schalk AM, Mondelaers V, De Moerloose B (2022). Novel Insights on the Use of L-Asparaginase as an efficient and safe anti-cancer therapy. Cancers.

[219] Krall AS, Mullen PJ, Surjono F, Momcilovic M, Schmid EW, Halbrook CJ (2021). Asparagine couples mitochondrial respiration to ATF4 activity and tumor growth. Cell Metab.

[220] Gwinn DM, Lee AG, Briones-Martin-Del-Campo M, Conn CS, Simpson DR, Scott AI (2018). Oncogenic KRAS regulates amino acid homeostasis and asparagine biosynthesis via ATF4 and alters sensitivity to L-Asparaginase. Cancer Cell.

[221] Qiu F, Huang J, Sui M (2015). Targeting arginine metabolism pathway to treat arginine-dependent cancers. Cancer Lett.

[222] Holtsberg FW, Ensor CM, Steiner MR, Bomalaski JS, Clark MA (2002). Poly(ethylene glycol) (PEG) conjugated arginine deiminase: effects of PEG formulations on its pharmacological properties. J Controlled Release.

[223] Steggerda SM, Bennett MK, Chen J, Emberley E, Huang T, Janes JR (2017). Inhibition of arginase by CB-1158 blocks myeloid cell-mediated immune suppression in the tumor microenvironment. J Immunother Cancer.

[224] Yao S, Janku F, Subbiah V, Stewart J, Patel SP, Kaseb A (2021). Phase 1 trial of ADI-PEG20 plus cisplatin in patients with pretreated metastatic melanoma or other advanced solid malignancies. Br J Cancer.

[225] Jeon H, Kim JH, Lee E, Jang YJ, Son JE, Kwon JY (2016). Methionine deprivation suppresses triple-negative breast cancer metastasis in vitro and in vivo. Oncotarget.

[226] Durando X, Farges M-C, Buc E, Abrial C, Petorin-Lesens C, Gillet B (2010). Dietary methionine restriction with FOLFOX regimen as first line therapy of metastatic colorectal cancer: a feasibility study. Oncology.

[227] Sanderson SM, Gao X, Dai Z, Locasale JW (2019). Methionine metabolism in health and cancer: a nexus of diet and precision medicine. Nat Rev Cancer.

[228] Maddocks ODK, Athineos D, Cheung EC, Lee P, Zhang T, van den Broek NJF (2017). Modulating the therapeutic response of tumours to dietary serine and glycine starvation. Nature.

[229] Viana LR, Tobar N, Busanello ENB, Marques AC, de Oliveira AG, Lima TI (2019). Leucine-rich diet induces a shift in tumour metabolism from glycolytic towards oxidative phosphorylation, reducing glucose consumption and metastasis in Walker-256 tumour-bearing rats. Sci Rep..

[230] Ishak Gabra MB, Yang Y, Li H, Senapati P, Hanse EA, Lowman XH (2020). Dietary glutamine supplementation suppresses epigenetically-activated oncogenic pathways to inhibit melanoma tumour growth. Nat Commun.

[231] Yue T, Li J, Zhu J, Zuo S, Wang X, Liu Y (2023). Hydrogen sulfide creates a favorable immune microenvironment for colon cancer. Cancer Res.

[232] Fang L, Hao Y, Yu H, Gu X, Peng Q, Zhuo H (2023). Methionine restriction promotes cGAS activation and chromatin untethering through demethylation to enhance antitumor immunity. Cancer Cell.

[233] Kim J, DeBerardinis RJ (2019). Mechanisms and implications of metabolic heterogeneity in cancer. Cell Metab.

[234] Varghese S, Pramanik S, Williams LJ, Hodges HR, Hudgens CW, Fischer GM (2021). The Glutaminase Inhibitor CB-839 (Telaglenastat) enhances the antimelanoma activity of T-Cell-Mediated immunotherapies. Mol Cancer Ther.

[235] Han T, Guo M, Zhang T, Gan M, Xie C, Wang JB (2017). A novel glutaminase inhibitor-968 inhibits the migration and proliferation of non-small cell lung cancer cells by targeting EGFR/ERK signaling pathway. Oncotarget.

